# Proteomics identifies potential immunological drivers of postinfection brain atrophy and cognitive decline

**DOI:** 10.1038/s43587-024-00682-4

**Published:** 2024-08-14

**Authors:** Michael R. Duggan, Zhongsheng Peng, Pyry N. Sipilä, Joni V. Lindbohm, Jingsha Chen, Yifei Lu, Christos Davatzikos, Guray Erus, Timothy J. Hohman, Shea J. Andrews, Julián Candia, Toshiko Tanaka, Cassandra M. Joynes, Chelsea X. Alvarado, Mike A. Nalls, Jenifer Cordon, Gulzar N. Daya, Yang An, Alexandria Lewis, Abhay Moghekar, Priya Palta, Josef Coresh, Luigi Ferrucci, Mika Kivimäki, Keenan A. Walker

**Affiliations:** 1 Laboratory of Behavioral Neuroscience, National Institute on Aging, National Institutes of Health, Baltimore, MD USA; 2 Clinicum, Department of Public Health, University of Helsinki, Helsinki, Finland; 3 Finnish Institute of Occupational Health, Helsinki, Finland; 4 Broad Institute of the MIT and Harvard University, The Klarman Cell Observatory, Cambridge, MA USA; 5 Brain Sciences, University College London, London, UK; 6 Department of Epidemiology, Johns Hopkins University Bloomberg School of Public Health, Baltimore, MD USA; 7 Department of Epidemiology, University of North Carolina at Chapel Hill, Chapel Hill, NC USA; 8 Department of Radiology, University of Pennsylvania, Philadelphia, PA USA; 9 Vanderbilt Memory and Alzheimer’s Center, Vanderbilt University Medical Center, Nashville, TN USA; 10 Vanderbilt Genetics Institute, Vanderbilt University Medical Center, Nashville, TN USA; 11 Department of Psychiatry and Behavioral Sciences, University of California San Francisco, San Francisco, CA USA; 12 Translational Gerontology Branch, National Institute on Aging, National Institutes of Health, Baltimore, MD USA; 13 Center for Alzheimer’s and Related Dementias, National Institutes of Health, Bethesda, MD USA; 14 DataTecnica LLC, Washington, DC USA; 15 Laboratory of Neurogenetics, National Institute on Aging, National Institutes of Health, Bethesda, MD USA; 16 Department of Neurology, Johns Hopkins University School of Medicine, Baltimore, MD USA; 17 Department of Neurology, University of North Carolina at Chapel Hill, Chapel Hill, NC USA

**Keywords:** Cognitive ageing, Molecular neuroscience

## Abstract

Infections have been associated with the incidence of Alzheimer disease and related dementias, but the mechanisms responsible for these associations remain unclear. Using a multicohort approach, we found that influenza, viral, respiratory, and skin and subcutaneous infections were associated with increased long-term dementia risk. These infections were also associated with region-specific brain volume loss, most commonly in the temporal lobe. We identified 260 out of 942 immunologically relevant proteins in plasma that were differentially expressed in individuals with an infection history. Of the infection-related proteins, 35 predicted volumetric changes in brain regions vulnerable to infection-specific atrophy. Several of these proteins, including PIK3CG, PACSIN2, and PRKCB, were related to cognitive decline and plasma biomarkers of dementia (Aβ_42/40_, GFAP, NfL, pTau-181). Genetic variants that influenced expression of immunologically relevant infection-related proteins, including ITGB6 and TLR5, predicted brain volume loss. Our findings support the role of infections in dementia risk and identify molecular mediators by which infections may contribute to neurodegeneration.

## Abstract (web summary)

This study reveals how infections that increase long-term dementia risk can contribute to longitudinal brain volume loss and regulate immunological proteins in plasma, and which of these proteins may drive infection-specific neurodegeneration.

## Main

A history of severe infections has been associated with increased risk for dementia and neurodegenerative diseases^1–5^, yet the mechanisms by which infections may contribute to this increased risk remain poorly understood. Cross-sectional neuroimaging studies indicate that acute viral and bacterial infections can be accompanied by brain volume loss^6^. Declines in gray matter thickness and total brain volume have been reported 6 months after severe acute respiratory syndrome-related coronavirus 2 (SARS-CoV-2) infection^7^, and we recently found accelerated white matter atrophy among individuals with a history of symptomatic herpetic infections^8^. Conversely, brain atrophy in aviremic people living with human immunodeficiency virus (HIV) may not persist over time^9,10^ and another study found no longitudinal evidence of herpes simplex virus-1-mediated cognitive decline or whole-brain atrophy among carriers of familial Alzheimer disease (AD) mutations^11^.

Systemic infections may influence dementia risk and neurodegeneration by triggering an acute inflammatory response or reshaping the host immune system, as in the case of chronic inflammation^12^. In response to immune insults, such as pathogens and tissue damage, changes in circulating inflammatory proteins can influence brain health through a variety of mechanisms, including their interactions with target cells in the central nervous system (CNS)^13^. For example, elevated cytokine signaling after SARS-CoV-2 infection can result in neuroinflammation and post-acute sequelae despite its low or absent copy numbers in the CNS^14,15^. Increases in plasma inflammatory markers among cognitively normal adults are associated with reduced brain volumes and cognitive performance, and greater dementia risk decades later^16–18^. Although several studies have tied select immune markers (for example, tumor necrosis factor (TNF), interleukin (IL)-1β) to preceding inflammatory events and ensuing cognitive performance^19,20^, it remains unknown how infections relate to an array of immunological proteins, and which of these proteins may predict changes in brain regions vulnerable to infection-specific atrophy.

In the present study, we used multiple large cohorts to examine how past infection diagnoses relate to changes in brain volumes over time and risk for all-cause dementia, AD dementia, and vascular dementia (VaD). After investigating the associations of infection history with brain volume loss and dementia risk, we used large-scale proteomics in the Baltimore Longitudinal Study of Aging (BLSA) to identify a subset of immunologically relevant, infection-related proteins in plasma that predict changes in brain regions vulnerable to infection-specific atrophy. Several of these, including PIK3CG (phosphatidylinositol-4,5-bisphosphate 3-kinase catalytic subunit γ), PACSIN2 (protein kinase C and casein kinase substrate in neurons protein 2), and PRKCB (protein kinase C β type), were also associated with longitudinal cognitive performance and plasma biomarkers of AD pathology (amyloid beta (Aβ)_42/40_, phosphorylated tau (pTau)-181), neuronal injury (neurofilament light chain (NfL)) or reactive astrogliosis (glial fibrillary acidic protein (GFAP)). Last, we found genetic variants that influenced expression of immunologically relevant, infection-related proteins, including integrin subunit β 6 (ITGB6) and toll-like receptor (TLR) 5, which also predicted brain volume loss in the BLSA and in an independent cohort using two-sample Mendelian randomization (MR).

## Results

### Infection history is associated with brain volume loss

**Fig. 1:**
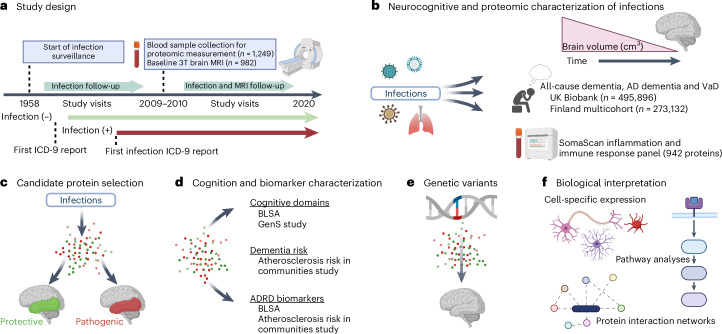
Study design. **a**, BLSA participants were classified according to the presence or absence of infection diagnoses using ICD-9 codes collected at study visits as early as 1958. Repeated 3T MRI scans were initiated in 2009–2010. Blood samples were collected at the initial 3T MRI scan and, for a subset of participants, at the time of a first PET scan as part of a separate study. **b**, Analyses examined how infection diagnoses are associated with brain volume changes over time and an immunological plasma proteome in the BLSA, as well as risk of all-cause dementia, AD dementia and VaD in the UK Biobank and a Finnish multicohort sample (the FPS study, the HeSSup study, and the STW study). **c**, Candidate proteins were selected if they were associated with an infection and related to changes in brain regions vulnerable to infection-specific atrophy, and were defined as protective or pathogenic, depending on whether they predicted preserved or reduced longitudinal brain volumes, respectively. **d**, Candidate proteins were related to longitudinal performance across five cognitive domains (the BLSA), cross-sectional performance across five cognitive domains (the GenS study), dementia risk (the ARIC study), and ADRD biomarkers (Aβ_42/40_, GFAP, NfL, pTau-181; the BLSA and the ARIC study). **e**, Genetic variants that influenced candidate protein levels were associated with changes in brain volumes in the BLSA and an external cohort (the ENIGMA consortium). **f**, The biological implications and functional relevance of candidate proteins were assessed using a variety of complementary analytical tools and open-source databases. All panels were created with BioRender.

Using data from the BLSA, we examined standardized longitudinal brain volume changes between participants with a history of a specific infection and participants without a history of such an infection (for example, influenza versus noninfluenza) (Fig. 1). Infections were classified a priori into categories using the *International Classification of Diseases*, 9th revision (ICD-9) codes^1^, and included influenza, pneumonia, tuberculosis, candidiasis/fungal, miscellaneous bacterial infections, gastrointestinal infections, sexually transmitted infections, human herpes virus (HHV) infections, viral hepatitis, miscellaneous viral infections, upper respiratory tract infections (URTIs), lower respiratory tract infections (LRTIs), skin and subcutaneous infections, urinary tract infections, and ‘other’ infections (Supplementary Table 1). A total of 982 cognitively normal participants (age 65.4 years (s.d. = 14.9 years); 55.2% female; 66.9% white) were included in these analyses (Supplementary Table 2 and Supplementary Fig. 1). The average time between the date of any infection diagnosis and baseline scan was 16 years (median: 13.9, interquartile range (IQR) = 8.2, 22.6) (Supplementary Table 3). The average follow-up time for magnetic resonance imaging (MRI) analyses was 5.3 years (median: 5.0, IQR = 3.9, 7.1) with an average of 3.4 (s.d. = 1.5) scans per participant in longitudinal analyses (range: 2–10). Among participants with and without infections related to brain volume changes, mean follow-up times were similar. Less than half (42.9%; *n* = 421) of participants exhibited a history of no infection diagnoses and 10.1% (*n* = 108) exhibited a history of two or more infection diagnoses. Primary regions of interest (ROIs) included total brain, gray matter, white matter and lobar volumes, as well as an AD-signature region volume^21,22^. Follow-up analyses were performed on lobar white/gray matter volumes if an infection was significantly associated with a primary ROI.

**Fig. 2:**
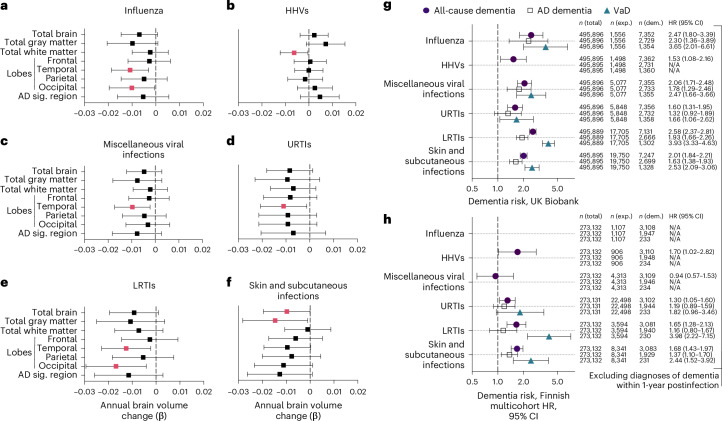
Annual changes in brain volumes and dementia risk associated with infections. **a**–**f**, Forest plots showing the associations of brain volume loss over time with influenza (**a**), HHVs (**b**), miscellaneous viral infections (**c**), URTIs (**d**), LRTIs, (**e**) and skin and subcutaneous infections (**f**) in the BLSA. Adjusted differences in annual changes of standardized brain volumes associated with a history of a given infection (β) were derived from linear mixed-effect models (*n* = 982) adjusted for intracranial volume, baseline age, sex, race, education, *APOEε4*, a comorbidity index (that is, obesity, hypertension, diabetes, cancer, ischemic heart disease, chronic heart failure, chronic kidney disease and chronic obstructive pulmonary disease) and two-way interactions of covariates with time. Pink squares reflect statistically significant associations. The AD signature region was the combined volume of the hippocampus, parahippocampal gyrus, entorhinal cortex, posterior cingulate gyrus, precuneus and cuneus. **g**,**h**, Additional forest plots showing the associations of infections with risk of all-cause dementia, AD dementia and VaD in the UK Biobank (**g**) and the Finnish multicohort sample (**h**; the FPS study, the HeSSup study and the STW study) after excluding dementia cases documented within 1 year postinfection. Columns report frequencies of the total sample, individuals exposed to a given infection and individuals diagnosed with dementia. Filled-in shapes reflect statistically significant associations. Data are presented as hazard ratios (HRs) and 95% confidence intervals (CIs). N/A indicates insufficient sample size to assess dementia risk. Adjusted differences in dementia risk associated with history of a given infection were derived from Cox proportional hazards regression models adjusted for age, sex and socioeconomic status. All-cause dementia included participants with a diagnosis of AD dementia, VaD, Parkinson’s disease dementia, frontotemporal dementia and other, less common dementia diagnoses (for example, unspecified dementia). Statistical significance was defined at a two-sided *P* < 0.05 without adjustment for multiple comparisons. The exact *P* values are presented in the source data files of Supplementary Tables 4, 7 and 9. AD sig, AD signature; exp., exposed; dem, dementia.

**Extended Data Fig. 1:**
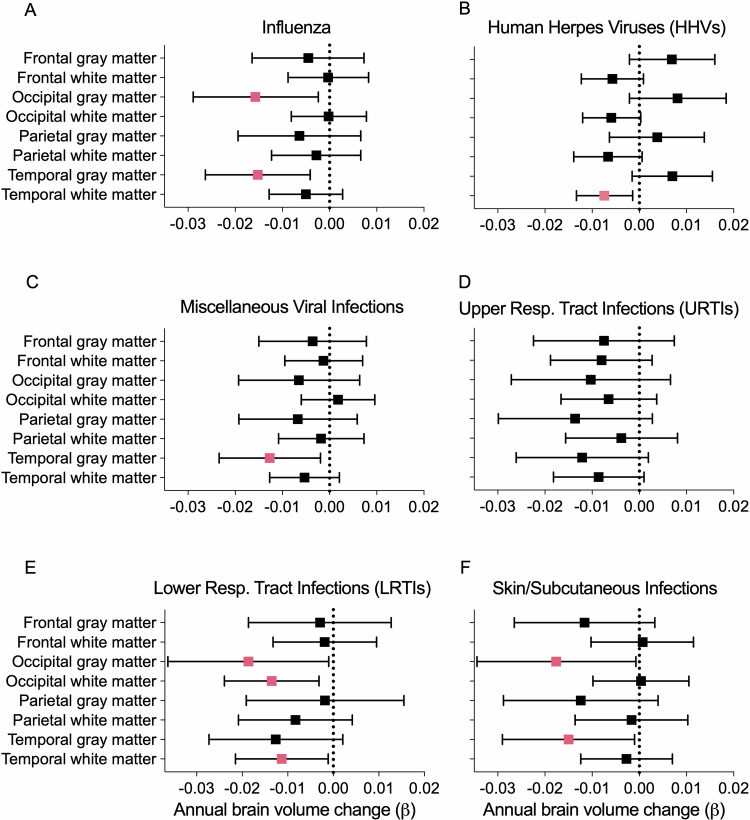
Annual changes in lobar white and gray matter volumes associated with infections. Forest plots show the associations of brain volume loss over time with **a** influenza **b** human herpes viruses **c** miscellaneous viral infections **d** upper respiratory tract infections **e** lower respiratory tract infections and **F** skin/subcutaneous infections in the Baltimore Longitudinal Study of Aging. Data are presented as beta coefficients and 95% confidence intervals. Pink squares reflect statistically significant associations. Adjusted differences in annual changes of standardized brain volumes associated with history of a given infection (β) were derived from linear mixed-effects models (n = 982) adjusted for intracranial volume, baseline age, sex, race, education, *APOEε4*, a comorbidity index (that is, obesity, hypertension, diabetes, cancer, ischemic heart disease, chronic heart failure, chronic kidney disease, and chronic obstructive pulmonary disease), and two-way interactions of covariates with time. Statistical significance was defined at two-sided p < 0.05 without adjustment for multiple comparisons. The exact p-values are presented in the source data files, Supplementary Table 4.

Of the 15 infections examined, 6 were associated with accelerated brain volume loss, predominantly in temporal gray and/or white matter regions (Fig. 2 and Supplementary Table 4). Follow-up analyses revealed that influenza-related volume loss was specific to temporal and occipital lobe gray matter (Fig. 2a and Extended Data Fig. 1a). Consistent with published findings^8^, declines in white matter volumes linked to herpetic infections (Fig. 2b) were localized to the temporal lobe (Extended Data Fig. 1b). Accelerated atrophy in the temporal lobe tied to miscellaneous viral infections was specific to gray matter (Fig. 2c and Extended Data Fig. 1c). URTIs were similarly linked to accelerated loss in total temporal lobe volume, but such decreases were not specific to either white or gray matter (Fig. 2d and Extended Data Fig. 1d). LRTI-related volume loss in the temporal lobe was exclusive to white matter, whereas decreases in the occipital lobe were evident in both white and gray matter (Fig. 2e and Extended Data Fig. 1e). Along with reduced total brain volume, the gray matter atrophy related to skin and subcutaneous infections was localized to the temporal and occipital lobes (Fig. 2f and Extended Data Fig. 1f). Although associations between infection history and brain volume changes were no longer statically significant after false recovery rate (FDR) correction, such correction may not be appropriate because of the inherent interdependence of the outcomes (for example, total gray matter loss is related to total brain volume loss). A history of any of the examined infections and total frequency of infections were not associated with volume changes. Despite limited power, sensitivity analyses restricting the comparison group to participants with a history of no infections (for example, influenza versus no history of any infection) showed consistent atrophy linked to influenza, herpes viruses, miscellaneous viral infections and LRTIs, with URTI- and skin and subcutaneous infection-related atrophy remaining marginally significant (*P* ≤ 0.10) (Supplementary Table 4). For primary ROIs, we did not find differences at baseline and results were similar after adjusting for total infection frequency (Supplementary Tables 4 and 5). These findings suggest that specific infections may be associated with accelerated brain volume loss, particularly in temporal regions.

### Infection history is a risk factor for dementia

**Extended Data Fig. 2:**
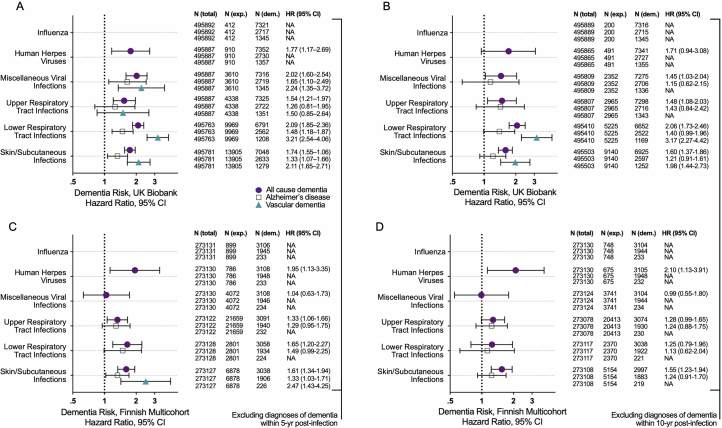
Dementia risk associated with infections. Forest plots show the associations of infections with risk of all-cause, Alzheimer’s disease, and vascular dementias in the UK Biobank after excluding dementia cases documented within **a** 5-yr and **b** 10-yr post-infection. Additional forest plots show the associations of infections with risk of all-cause, Alzheimer’s disease, and vascular dementias in the Finnish multicohort sample (the Finnish Public Sector study, the Health, and Social Support study, and the Still Working study) after excluding dementia cases documented within **c** 5-yr and **d** 10-yr post-infection. Columns report frequencies of the total sample, individuals exposed to a given infection, and individuals diagnosed with dementia. Data are presented as hazard ratios and 95% confidence intervals. NA indicates insufficient sample size to assess dementia risk. Filled in shapes reflect statistically significant associations. Adjusted differences in dementia risk associated with history of a given infection (hazard ratios) were derived from Cox proportional hazards regression models adjusted for age, sex, and socioeconomic status. All-cause dementia included participants with a diagnosis of Alzheimer’s disease dementia, vascular dementia, Parkinson’s disease dementia, frontotemporal dementia, and other, less commonly specified dementia diagnoses (for example, unspecified dementia). Statistical significance was defined at two-sided p < 0.05 without adjustment for multiple comparisons. The exact p-values are presented in the source data files, Supplementary Tables 7, 9.

Using UK Biobank data (*n* = 495,896; Supplementary Table 6 and Supplementary Fig. 2), we examined associations between infections linked to brain volume loss in the BLSA and risk for incident all-cause dementia, AD dementia, and VaD. After excluding dementia cases documented within 1 year postinfection (that is, to reduce the risk of ascertainment and reverse causation biases) and adjusting for demographic factors (age, sex, socioeconomic status), all infections linked to brain volume loss in the BLSA were also associated with increased risk of all-cause dementia (Fig. 2g). Although four out of five of these infections were also related to AD dementia and five out of five were related to VaD, risk was particularly elevated for VaD. With few exceptions, the results were similar after adjusting for physiological/lifestyle factors (body mass index (BMI), hypertension, diabetes, *APOE* genotype, alcohol consumption, smoking) and excluding dementia cases documented within 5 years postinfection; however, after applying a 10-year exclusion criterion, risk for AD dementia was attenuated whereas risk of all-cause dementia and VaD persisted (Supplementary Table 7 and Extended Data Fig. 2a,b). Insufficient sample size prevented the assessment of some infections with etiology-specific dementia risk (for example, HHVs/VaD).

Using the Finnish multicohort sample (*n* = 273,132; Supplementary Table 8 and Supplementary Fig. 3), along with similar exclusion criteria and demographic adjustments, we found that four out of five infections linked to brain volume loss in the BLSA were also associated with increased risk of incident all-cause dementia, with miscellaneous viral infections (related to increased dementia risk in the UK Biobank only) being the exception (Fig. 2h). One and two of these three infections were associated with elevated risk of AD dementia and VaD, respectively. Although skin and subcutaneous infections were related to both AD dementia and VaD, risk was particularly elevated for VaD. Results were similar after adjusting for physiological factors (hypertension, diabetes) and excluding dementia cases documented within 5 years postinfection. After applying the 10-year exclusion criterion, insufficient sample size prevented VaD assessment, but AD dementia risk was mitigated whereas all-cause dementia risk persisted for herpetic as well as skin and subcutaneous infections (Supplementary Table 9 and Extended Data Fig. 2c,d).

### Infection history is related to an altered immune proteome

Next, we examined the plasma immune proteomic signature of infections linked to brain atrophy using the SomaScan v.4.1 Inflammation and Immune Response Panel (942 proteins; Supplementary Table 10). A total of 1,184 cognitively normal BLSA participants were included in these analyses and the average time between the date of infection diagnoses and blood sample collection was 13.8 years (median: 11.9, IQR = 4.4, 18.7) (Supplementary Tables 11 and 12 and Supplementary Fig. 4). In our proteomic sample, 63.2% (*n* = 749) of participants illustrated a history of any infection and 16.6% (*n* = 197) exhibited a history of an infection linked to longitudinal brain atrophy.

**Fig. 3:**
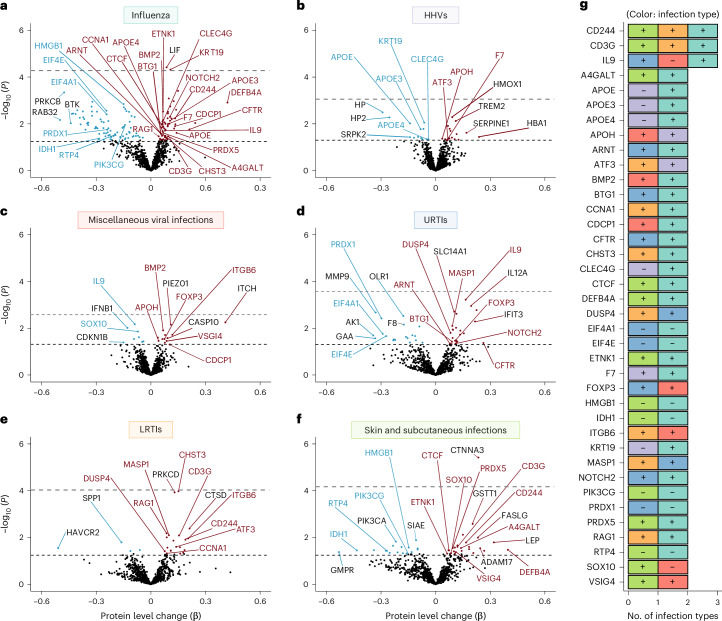
Differences in immunological plasma proteins (942 proteins; SomaScan Inflammation and Immune Response Panel) associated with infections in the BLSA. **a**–**f**, Volcano plots showing the differences in protein levels associated with influenza (**a**), HHVs (**b**), miscellaneous viral infections (**c**), URTIs (**d**), LRTIs, (**e**) and skin and subcutaneous infections (**f**). Adjusted differences in log_2_(protein levels) associated with history of a given infection (β) were derived from multiple linear regression models (*n* = 1,184) adjusted for age, sex, race, education, *APOEε4*, eGFR-creatinine, and a comorbidity index (that is, obesity, hypertension, diabetes, cancer, ischemic heart disease, chronic heart failure, chronic kidney disease, and chronic obstructive pulmonary disease). Proteins above the dashed horizontal black line were statistically significant (uncorrected *P* < 0.05), with red and blue dots indicating positive and negative associations, respectively. The FDR-corrected *P*-value threshold is indicated by the dashed gray line. Red and blue labels represent proteins associated with two or more infections, whereas black labels indicate proteins uniquely associated with a given infection. **g**, A clustered bar graph showing proteins associated with two or more infections. Adjusted differences in log_2_(protein levels) associated with history of a given infection were derived from multiple linear regression models adjusted for the aforementioned covariates. The (+) and (−) indicate that higher or lower protein levels were associated with a given infection. Statistical significance was defined at two-sided *P* < 0.05 without adjustment for multiple comparisons. The exact *P* values are presented in the source data files of Supplementary Table 13.

Influenza was associated with the highest number of differentially expressed proteins (141 proteins) and miscellaneous viral infections were associated with the fewest (21 proteins) (Fig. 3a–f and Supplementary Table 13). Among proteins uniquely associated with specific infections, we observed lower levels of MME (aka neprilysin; primary Aβ degradation enzyme) with influenza, higher levels of TREM2 (myeloid receptor) with HHVs, higher levels of IFIT3 (interferon-induced antimicrobial peptide) with URTIs, and higher levels of ADAM17 (TREM2-sheddase) with skin and subcutaneous infections. Among 260 proteins associated with at least 1 infection, many were previously linked to influenza, Zika and/or SARS-CoV-2 infection(s), including 54 out of 74 (73%) measured on the commonly used SomaScan v.1 platforms and 93 of 260 (36%) measured on any SomaScan platform (Supplementary Table 14). Among proteins related to multiple infections, 30 out of 38 were associated with influenza, including total APOE, APOE3, APOE4 (apolipoproteins), DEFB4A (an antimicrobial peptide) and RAG1 (a VDJ recombination activator) (Fig. 3g). Two proteins associated with influenza (LIF, KRT19 (which was also related to HHVs)) and one protein associated with skin and subcutaneous infections (CTNNA3) remained statistically significant at *P* < 0.05 after FDR correction. In addition to their associations with accelerated brain atrophy and increased dementia risk, these results indicate that a history of infection is associated with differences in immunologically relevant proteins, many of which are associated with multiple infections.

### Infection-related proteins predict brain atrophy

Using BLSA proteomic and longitudinal brain MRI data, we then identified which infection-related proteins were associated with changes in brain volumes over time in regions vulnerable to infection-specific atrophy. A total of 977 participants were included in these analyses (Supplementary Table 15 and Supplementary Fig. 5). The average follow-up was 5.3 years (median: 5.0, IQR = 3.9, 7.0) with an average of 3.4 (s.d. = 1.5) MR scans (range: 2–10) per participant in longitudinal analyses.

**Fig. 4:**
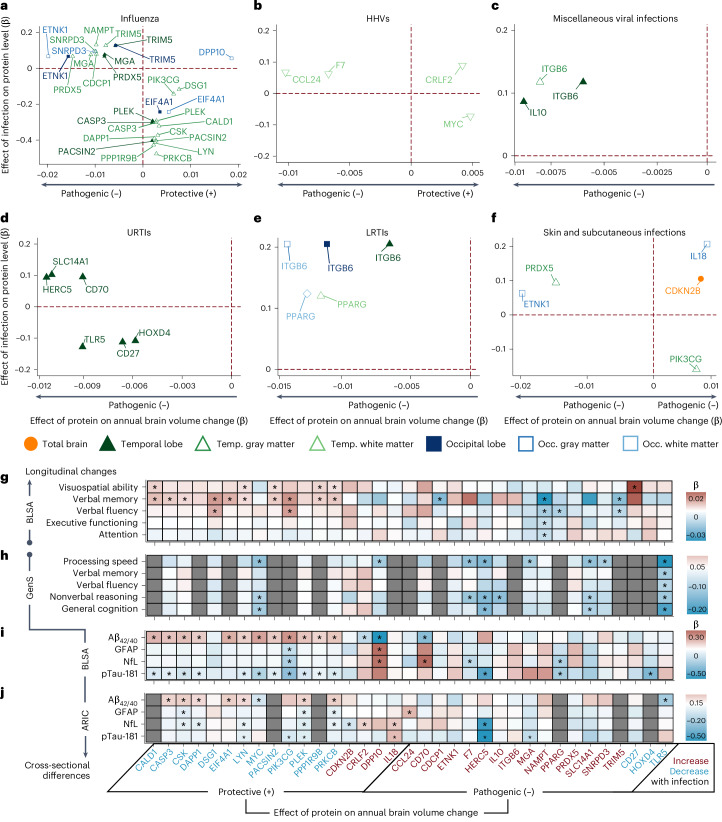
Candidate proteins relate to changes in brain regions vulnerable to infection-specific atrophy, longitudinal cognitive performance, plasma biomarkers and cognitive performance. **a**–**f**, Scatterplots showing how differences in protein levels associated with specific infections (*y* axis) relate to a given protein’s longitudinal effect on brain regions vulnerable to infection-specific atrophy (*x* axis) in the BLSA, specifically for influenza (**a**), HHVs (**b**), miscellaneous viral infections (**c**), URTIs (**d**), LRTIs, (**e**) and skin and subcutaneous infections (**f**). Candidate proteins were defined as protective or pathogenic, depending on their associations with preserved or reduced longitudinal brain volumes. Differences in protein levels associated with infections (*y* axis, β) were derived from linear regression models (*n* = 1,184) adjusted for the aforementioned covariates, and differences in brain volumes changes related to protein levels (*x* axis, β) were derived from linear mixed-effect models (*n* = 977) adjusted for similar covariates plus intracranial volume and two-way interactions with time. All displayed associations are statistically significant. **g**, Heatmap showing associations of candidate proteins with longitudinal cognitive performance in the BLSA. Differences in annual changes of cognitive scores related to protein levels (β) were derived from linear mixed-effect models (*n* = 1,233) adjusted for the aforementioned covariates. **h**, Heatmap showing associations of candidate proteins with cognitive performance in the GenS cohort. Differences in cognitive scores related to protein levels (β) were derived from linear mixed-effect models (*n* = 1,065) corrected for relatedness across individuals, age, sex, depression, study site, and storage time. **i**, Heatmap showing associations of candidate proteins with plasma biomarkers in the BLSA. Differences in biomarkers related to protein levels (β) were derived from linear regression models (Aβ_42/40_, GFAP and NfL, *n* = 757; pTau-181, *n* = 674) adjusted for the aforementioned BLSA covariates plus eGFR-creatinine. **j**, Heatmap showing associations of candidate proteins with plasma biomarkers in the ARIC study. Differences in biomarkers related to protein levels (β) were derived from linear regression models (*n* = 1,419) adjusted for age, sex, race center, education, *APOE*ε4, eGFR-creatinine, and cardiovascular risk factors. ^*^Statistically significant (uncorrected *P* < 0.05). Statistical significance was defined at two-sided *P* < 0.05 without adjustment for multiple comparisons. The exact *P* values are presented in the source data files of Supplementary Tables 13, 16, 19, 20, 24 and 26. Temp. Temporal; Occ., Occipital.

**Extended Data Fig. 3:**
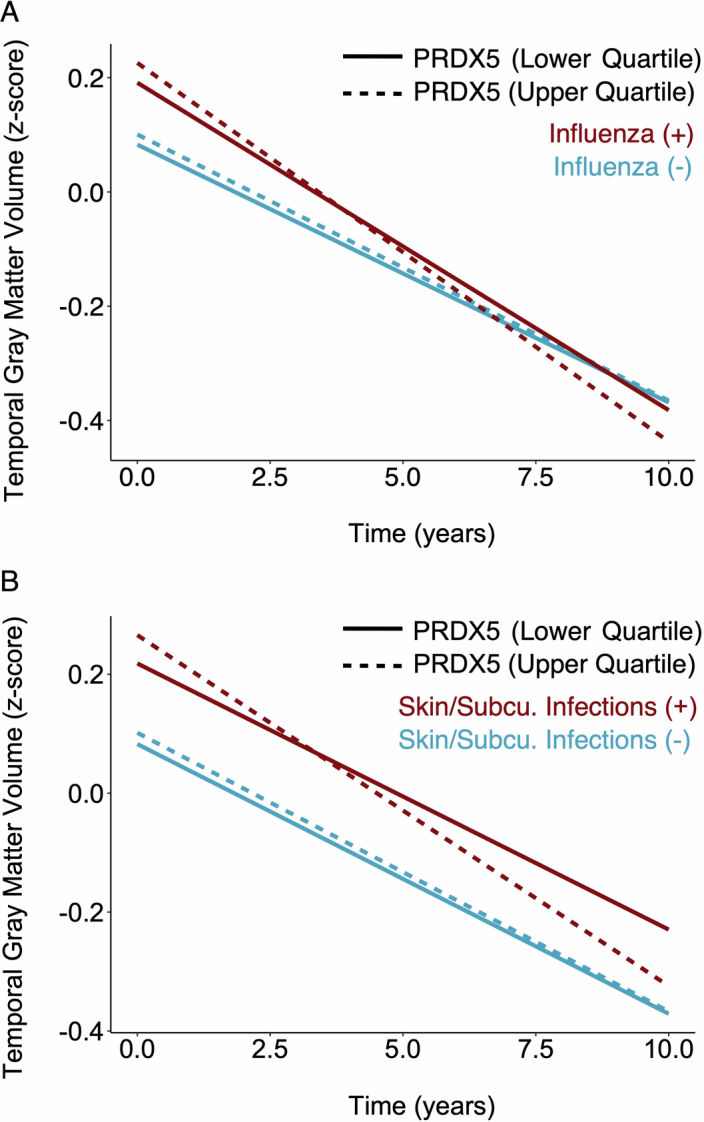
Annual changes in brain volumes associated with candidate proteins as a function of infection. Line graphs display annual changes in temporal gray matter volumes associated with PRDX5 levels as a function of **a** influenza and **b** skin/subcutaneous infections in the Baltimore Longitudinal Study of Aging. To improve interpretation, the effects of PRDX5 are displayed based on lower/upper PRDX5 quartiles (continuous PRDX5 levels were used in analyses). Adjusted differences in annual changes of standardized brain volumes associated with history of a given infection were derived from linear mixed-effects models (n = 1,184) adjusted for intracranial volume, baseline age, sex, race, education, *APOEε4*, a comorbidity index (that is, obesity, hypertension, diabetes, cancer, ischemic heart disease, chronic heart failure, chronic kidney disease, and chronic obstructive pulmonary disease), and two-way interactions of covariates with time, as well as two-way and three-way interaction terms (infection*protein, infection*protein*time) to examine whether an infection diagnosis modified the association of protein level with longitudinal brain volume change. The displayed associations reflect statistically significant relationships. Statistical significance was defined at two-sided p < 0.05 without adjustment for multiple comparisons. The exact p-values are presented in the source data files, Supplementary Table 17.

Among the 260 proteins associated with at least one infection (Fig. 4a–f, *y* axis), we identified 35 that were also associated with brain volume changes (Fig. 4a–f, *x* axis), henceforth referred to as candidate proteins. Candidate proteins were further defined as protective or pathogenic, depending on whether they were associated with preserved or reduced longitudinal brain volumes, respectively. With few exceptions (for example, DPP10 with influenza*),* protective proteins were decreased among individuals with a prior infection, whereas pathogenic proteins were increased (Supplementary Table 16). We also examined whether the associations of candidate proteins with changes in brain volumes were conditional on infection diagnosis using models that incorporated three-way interaction terms (that is, infection × protein × time) (Supplementary Table 17). Among four protective proteins (PPP1R9B, CALD1, PLEK and DAPP1) and one pathogenic protein (PRDX5) that decreased and increased with influenza, respectively, the associations with brain volume loss were in the expected directions and more robust among participants with a history of influenza compared with those without. We found similar evidence of increased effects among individuals with past infection for one pathogenic protein (ITGB6) elevated with LRTIs, and another (PRDX5) increased with skin and subcutaneous infections (Extended Data Fig. 3). These results identified 35 candidate proteins, including a set of protective proteins that are decreased postinfection and a set of pathogenic proteins that are increased postinfection, by which infections may contribute to neurodegeneration, with the effects of several proteins magnified among infected individuals.

### Candidate proteins are associated with cognitive decline

Next, we used data from the BLSA to determine whether levels of candidate proteins were associated with changes in cognitive performance across five cognitive domains. A total of 1,233 cognitively normal participants were included in analyses (Supplementary Tables 18 and 19 and Supplementary Fig. 6). The average follow-up time was 6.4 years (median: 6.1, IQR = 4.0, 8.3) with an average of 4.3 (s.d. = 2.4) cognitive assessments (range: 2–22) per participant in longitudinal analyses. Among 13 protective proteins that were decreased postinfection, 10 were associated with preserved verbal memory performance over time, a subset of which were also related to preserved visuospatial or verbal fluency performance (Fig. 4g). Accelerated declines in performance were associated with four pathogenic proteins, including one (NAMPT) linked to decreased capacities across four cognitive domains. One pathogenic protein (CD27) was unexpectedly related to preserved visuospatial abilities.

**Extended Data Fig. 4:**
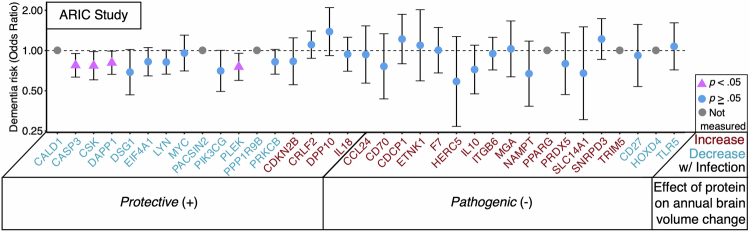
Dementia risk in the Atherosclerosis Risk in Communities (ARIC) study. A forest plot shows odds ratio of pre-existing all-cause dementia associated with candidate proteins. Adjusted odds of dementia risk were derived from binary logistic regression models (n = 4,743) adjusted for age, sex, race-center, education, APOEε4, eGFR-creatinine, and cardiovascular risk factors (BMI, diabetes, hypertension, and current smoking status). Pink circles reflect statistically significant associations. Data are presented as odds ratios and 95% confidence intervals. Statistical significance was defined at two-sided p < 0.05 without adjustment for multiple comparisons. The exact p-values are presented in the source data files, Supplementary Table 22.

We leveraged computed results from the Generation Scotland (GenS) cohort^23^ (*n* = 1,065) to determine whether levels of candidate proteins were associated with cross-sectional differences across five cognitive domains, some of which were assessed in the BLSA using similar tasks (Supplementary Table 20). Among the ten pathogenic proteins increased postinfection in the BLSA and measured in GenS, six were associated with lower cognitive performance, especially in processing speed and nonverbal reasoning (Fig. 4h). One pathogenic protein (TLR5) was related to lower performance across all domains, whereas another protective protein (MYC) was surprisingly related to lower performance in processing speed, nonverbal reasoning and general cognition. We also examined how candidate protein levels were related to pre-existing all-cause dementia using cross-sectional data from the Atherosclerosis Risk in Communities (ARIC) study (*n* = 4,743; Supplementary Table 21 and Supplementary Fig. 7). Four protective proteins (PLEK, DAPP1, CSK and CASP3) that decreased postinfection in the BLSA were significantly decreased in dementia cases in the ARIC study, with several other protective proteins (EIF4A1, DSG1, PIK3CG, PRKCB and LYN) showing similar trends (Extended Data Fig. 4 and Supplementary Table 22).

Together, these findings indicate that the direction of protective and pathogenic effects of candidate proteins are generally consistent with respect to changes in cognitive performance, and suggest that protective proteins decreased postinfection might otherwise be important for maintaining cognitive capacities over time.

### Candidate proteins are associated with dementia biomarkers

We next examined how candidate proteins related to plasma biomarkers of AD pathology (Aβ_42/40_ ratio, pTau-181) and neurodegeneration (GFAP, NfL) using data from the BLSA and the ARIC study. BLSA analyses of Aβ_42/40_, GFAP and NfL included 757 participants and pTau-181 analyses included 674 participants, all of whom were cognitively normal (Supplementary Tables 23 and 24 and Supplementary Fig. 8). In the BLSA, among 13 protective proteins decreased postinfection, 12 were associated with a significantly higher Aβ_42/40_ ratio (indicative of lower cortical amyloid) and 11 with lower levels of pTau-181 (Fig. 4i). However, two protective proteins were associated with a lower Aβ_42/40_ ratio, one of which (DPP10) was also related to increased NfL and GFAP. Of the pathogenic proteins, we found one protein (CD70) related to a lower Aβ_42/40_ ratio and elevated NfL, whereas several other proteins displayed unexpected negative associations with NfL and/or pTau-181. Among ten protective proteins that decreased postinfection in the BLSA and measured in the ARIC study (*n* = 1,419; Supplementary Tables 25 and 26 and Supplementary Fig. 9), eight were associated with lower levels of one or more plasma biomarkers, especially a higher Aβ_42/40_ ratio (indicative of lower cortical amyloid) (Fig. 4j). MYC showed divergent associations with the Aβ_42/40_ ratio across the BLSA and the ARIC study. Several protective proteins (IL-18, CCL24) that increased postinfection in the BLSA were related to higher NfL levels in the ARIC study, whereas two pathogenic proteins showed unexpected negative associations with pTau-181 (HERC5, MGA). Our results suggest that protective proteins decreased postinfection might otherwise be important for proximally mitigating CNS amyloidosis and tau phosphorylation, two early features of AD pathogenesis.

### Candidate protein genetic variation predicts brain atrophy

**Fig. 5:**
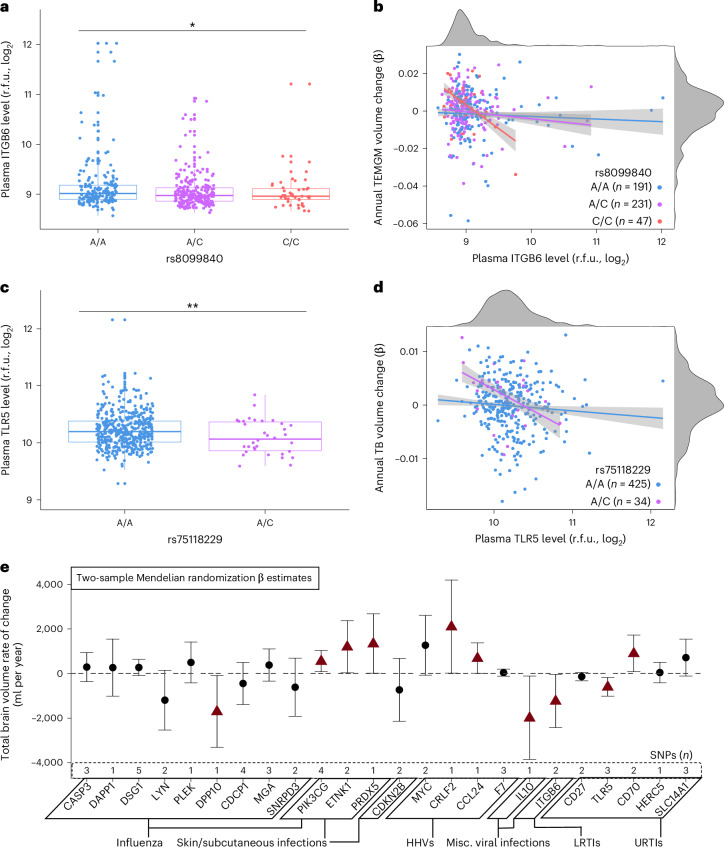
Genetic variants that influence expression of candidate proteins also relate to brain volume loss in the BLSA and an independent cohort, the ENIGMA consortium. **a**, A boxplot showing plasma levels of ITGB6 stratified by the rs8099840 genotype. **b**, A scatterplot showing the associations of plasma ITGB6 protein levels with rates of temporal gray matter (TEMGM) volume loss over time stratified by rs8099840 alleles. **c**, A boxplot of TLR5 plasma levels stratified by the rs75118229 genotype. **d**, A scatterplot showing the associations of plasma TLR5 protein levels with rates of total brain (TB) volume loss over time stratified by rs75118229 alleles. Adjusted differences in log_2_(protein levels) associated with a given SNP were derived from multiple linear regression models (*n* = 469) adjusted for covariates used in the pQTL discovery cohort, namely baseline age and sex. Adjusted differences in annual changes of standardized brain volumes associated with a given SNP were derived from linear mixed-effect models (*n* = 469) adjusted for similar covariates, along with intracranial volume and two-way interactions with time. Individual specific rates of brain volume loss (random effect of time) are displayed. Associations displayed in **b** and **d** reflect statistically significant relationships. ^*^Statistically significant (uncorrected *P* < 0.05); ^**^statistically significant (uncorrected *P* < 0.01). Box plots: median, 25–75th quartiles; whiskers: 1.5× the IQR. The pQTLs were obtained from deCODE Genetics and SNPs in the BLSA were used for analyses. **e**, A forest plot displaying the results of Mendelian randomization analyses that assessed the relationship between genetically determined plasma protein levels and changes in MRI-derived TB volumes. The results were derived from inverse variance-weighted or Wald’s ratio estimates. Plasma pQTLs were obtained from deCODE Genetics (*n* = 35,559). Longitudinal TB volume summary statistics were obtained from an ENIGMA consortium GWAS (*n* = 15,640). Data are presented as β coefficients and 95% CIs. Red triangles indicate statistically significant associations (uncorrected *P* < 0.05). Statistical significance was defined at two-sided *P* < 0.05 without adjustment for multiple comparisons. The exact *P* values are presented in the source data files of Supplementary Tables 29 and 30. Misc., miscellaneous; r.f.u., relative fluorescence units.

**Extended Data Fig. 5:**
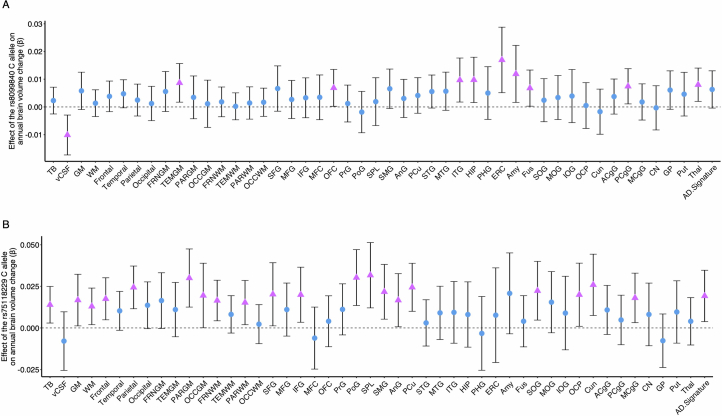
Annual changes in brain volumes associated with genetic variants. Forest plots show the associations of brain volume loss over time with **a** the rs8099840 C allele (which significantly influenced levels of ITGB6) and **b** the rs75118229 C allele (which significantly influenced levels of TLR5) in the Baltimore Longitudinal Study of Aging. Adjusted differences in annual changes of standardized brain volumes associated with a given SNP were derived from linear mixed-effects models (n = 469) adjusted for covariates used in the pQTL discovery cohort (deCODE Genetics), namely intracranial volume, baseline age and sex and two-way interactions of age and sex with time. Pink triangles reflect statistically significant associations (uncorrected *p* < 0.05). Data are presented as beta coefficients and 95% confidence intervals. Statistical significance was defined at two-sided p < 0.05 without adjustment for multiple comparisons. Key: TB, Total brain; vCSF, Ventricle; GM, Gray matter; WM, White matter; Frontal, Frontal lobe; Temporal, Temporal lobe; Parietal, Parietal lobe; Occipital, Occipital lobe; FRNGM, Frontal gray matter; TEMGM, Temporal gray matter; PARGM, Parietal gray matter; OCCGM, Occipital gray matter; FRNWM, Frontal white matter; TEMWM, Temporal white matter; PARWM, Parietal white matter; OCCWM, Occipital white matter; SFG, Superior frontal gyrus; MFG, Middle frontal gyrus; IFG, Inferior frontal gyrus; MFC, Medial frontal cortex; OFC, Orbitofrontal gyrus; PrG, Precentral gyrus; PoG, Postcentral gyrus; SPL, Superior parietal lobe; SMG, Supramarginal gyrus; AnG, Angular gyrus; PCu, Precuneus; STG, Superior temporal gyrus; MTG, Middle temporal gyrus; ITG, Inferior temporal gyrus; HIP, Hippocampus; PHG, Parahippocampal gyrus; ERC, Entorhinal cortex; Amy, Amygdala; Fus, Fusiform gyrus; SOG, Superior occipital gyrus; MOG, Middle occipital gyrus; IOG, Inferior occipital gyrus; OCP, Occipital pole; Cun, Cuneus; ACgG, Anterior cingulate gyrus; PCgG, Posterior cingulate gyrus; MCgG, Middle cingulate gyrus; CN, Caudate; GP, Global pallidum; Put, Putamen; Thal, Thalamus. The AD-signature region volume was the combined volume of hippocampus, parahippocampal gyrus, entorhinal cortex, posterior cingulate gyrus, precuneus, and cuneus. The exact p-values are presented in the source data files, Supplementary Table 29.

Last, we determined whether genetic variants that influence candidate protein levels in plasma relate to changes in brain volumes in the BLSA using protein quantitative trait loci (pQTLs) identified by the deCODE study (*n* = 35,559)^24^. Using candidate pQTLs as genetic instruments for 469 BLSA participants with SNP data^25^ (Supplementary Table 27 and Supplementary Fig. 10), we replicated SNP associations with plasma protein levels for 11 of the 24 candidate proteins with available pQTLs (at least one SNP per protein). Nearly all candidate protein genetic variants identified in the deCODE study and the subset replicated in BLSA were *trans*-pQTLs (Supplementary Tables 28 and 29). Genetic variants associated with decreased levels of pathogenic proteins ITGB6 (rs8099840; Fig. 5a,b), TLR5 (rs1403631, rs75118229; Fig. 5c,d), F7 (rs6046), HERC5 (rs406936) and MGA (rs704) were associated with preserved brain volumes over time, including in regions susceptible to infection-specific atrophy (Extended Data Fig. 5). These findings support a potentially causal or pleiotropic link between a subset of candidate proteins and brain atrophy. However, genetic variants that decreased levels of pathogenic proteins HERC5 (rs9332741), TLR5 (rs28427460), F7 (rs762635, rs3211727) and CD27 (rs16860992, rs1042464) were also associated with accelerated volume loss.

To determine whether genetic variants that influence candidate protein levels may also be associated with changes in brain volumes in an external cohort, we combined these candidate protein pQTLs with summary statistics from a genome-wide association study (GWAS) of brain volume change performed in the Enhancing Neuroimaging Genetics through Meta-Analyses (ENIGMA) consortium (*n* = 15,640)^26^ and applied two-sample MR. After pruning, 24 candidate proteins maintained at least one pQTL instrumental variable. We found evidence of a relationship between the levels of ten candidate proteins and changes in total brain volume (Fig. 5e). MR supported a protective effect of two proteins (PIK3CG and CRLF2) and the pathogenic effect another three (ITGB6, IL-10 and TLR5). Five proteins (PRDX5, ETNK1, CCL24, CD70 and DPP10) showed associations with brain volumes in MR that differed from those found in the BLSA. Results for proteins with more than two pQTLs derived using a weighted-median method provided further support for the effects of PIK3CG, TLR5 and CD70 on brain volume changes (Supplementary Tables 28, 30 and 31 and Supplementary Fig. 11). These findings suggest distal genetic variation modulates levels of several candidate proteins in plasma and that such modulation may be mechanistically relevant to neurodegeneration.

### Candidate protein characterization

**Fig. 6:**
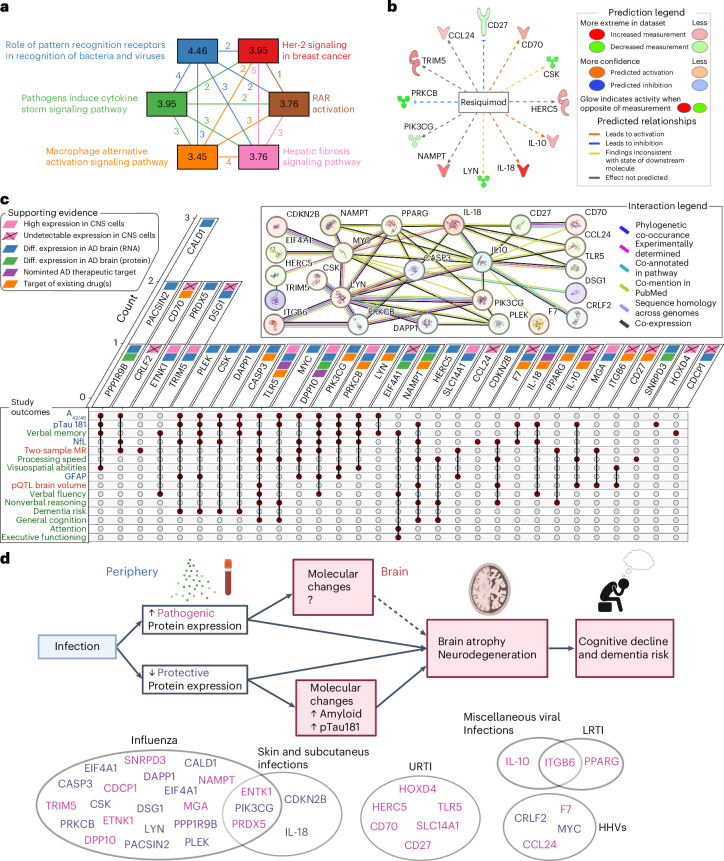
Pathway analysis and summary of evidence. **a**, A canonical pathway plot showing the top six biological pathways enriched for candidate proteins. Values in the nodes correspond to −log(Benjamini-Hochberg corrected) *P* values derived from Fisher’s exact tests. Values in between nodes correspond to frequencies of overlapping candidate proteins across respective biological pathways, with color coding used to improve interpretability. The results are derived from ingenuity pathway analysis (IPA). **b**, A radial plot showing resiquimod, an antiviral medication and the top upstream regulator of candidate proteins, as well as its predicted effects on specific candidate proteins. Shapes correspond to protein type (cytokine, enzyme, transmembrane, kinase). Results were derived from Fisher’s exact tests in IPA. **c**, An upset plot summarizing evidence for candidate proteins, including empirical results obtained from the current analyses, as well as protein–protein interaction networks (STRING; predicted protein conformations are depicted in each circular node), cell-specific expression patterns (Human Protein Atlas), expression levels (RNA, protein) in postmortem AD brain tissue (AMP-AD), nominated therapeutic targets (AMP-AD), and targets of known medications (Open Targets). **d**, Proposed model by which infections may contribute to increased risk for neurodegeneration. A history of specific infections is linked to increased levels of proteins that exert deleterious effects on brain volumes over time (pathogenic) and decreased levels of proteins associated with stable brain volumes (protective), which are indicated in corresponding circles for each infection type. Protective proteins may exert their effects through alterations to amyloidogenic pathways, as indicated by changes in plasma Aβ_42/40_ ratio and pTau-181, whereas pathogenic proteins may exert their effects through other mechanisms that require further elucidation. Modulation of the plasma immune proteome among individuals with a prior infection and subsequent changes in rates of brain atrophy over time may ultimately increase risk for cognitive decline and dementia. The exact *P* values are presented in the source data files of Supplementary Tables 32–39. Diff., differential; RAR, retinoic acid receptor. Panel **d** was created with BioRender.

**Extended Data Fig. 6:**
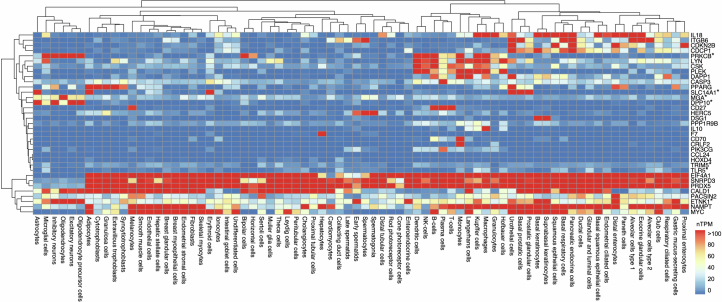
Expression in all cell types. Heatmap shows expression levels of genes encoding candidate proteins (cognate genes) across 76 available cell types based on single cell transcriptomics data sourced from the Human Protein Atlas. *Gene encoding candidate protein was highly expressed in at least one CNS cell type. Dendrograms reflect hierarchical clustering using Euclidean distances calculated from normalized Transcripts per Million. Key: nTPM, normalized Transcripts per Million. The exact p-values are presented in the source data files, Supplementary Table 35.

**Extended Data Fig. 7:**
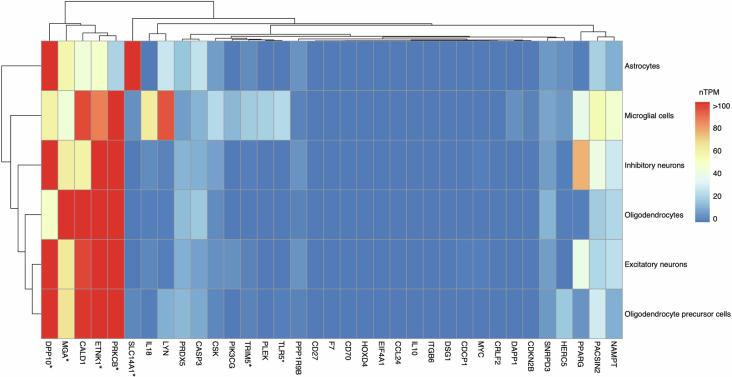
Expression in CNS cell types. Heatmap shows expression levels of genes encoding candidate proteins (cognate genes) across CNS cell types based on single cell transcriptomics data sourced from the Human Protein Atlas. *Gene encoding candidate protein was highly expressed in at least one CNS cell type. Dendrograms reflect hierarchical clustering using Euclidean distances calculated from normalized Transcripts per Million. Key: nTPM, normalized Transcripts per Million. The exact p-values are presented in the source data files, Supplementary Table 35.

**Extended Data Fig. 8:**
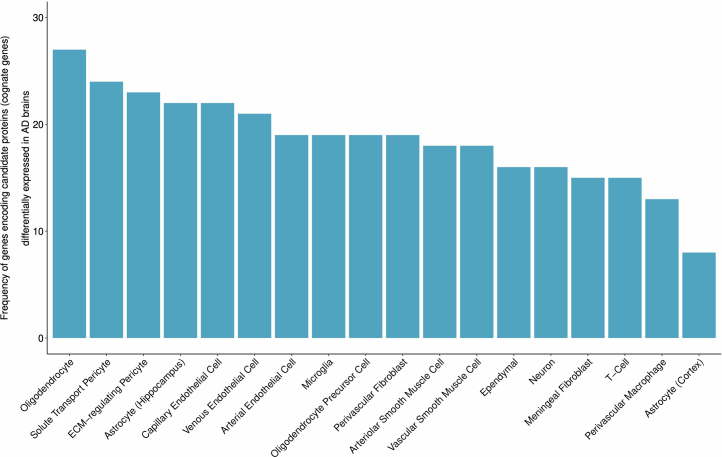
Expression in neurovascular cell types. A bar graph shows the frequency of genes encoding candidate proteins (cognate genes) differentially expressed in AD brains across 18 different neurovascular cell types. Results derived from Welch’s *t*-test that compared expression levels between AD and control participants. Key: AD, Alzheimer’s disease. The exact p-values are presented in the source data files, Supplementary Table 38.

Pathway analyses demonstrated that candidate proteins were enriched for pathogen-related signaling cascades (Fig. 6a, Supplementary Table 32 and Supplementary Figs. 12 and 13) and identified an antiviral medication (resiquimod) as their strongest upstream regulator (Fig. 6b and Supplementary Table 33). Protein interaction analyses revealed a robust network among candidate proteins, including co-expression patterns (Fig. 6c and Supplementary Table 34). The levels of seven cognate genes encoding candidate proteins were highly expressed in at least one CNS cell type, but another eleven were undetectable in CNS cells (Fig. 6c, Extended Data Figs. 6 and 7 and Supplementary Tables 35 and 36). Leveraging results obtained from comprehensive postmortem tissue collections, every cognate gene with available transcriptomic data was differentially expressed at the RNA level in AD brains, with another three also differentially expressed at the protein level (Fig. 6c and Supplementary Table 37). Among neurovascular cell types, patterns of AD-related expression were most apparent in pericytes, oligodendrocytes and astrocytes (Supplementary Table 38 and Extended Data Fig. 8). Four candidate proteins (IL-18, IL-10, TLR5, and DPP10) have been nominated as targets for AD treatment by the AMP-AD (Accelerating Medicine Partnership Program for AD) consortium (Fig. 6c and Supplementary Table 37). We also found 93 medications (primarily monoclonal antibodies) that modify levels of 13 candidate proteins, suggesting that such drugs may have the potential for repurposing in the treatment of neurodegeneration, particularly among individuals at increased risk for, or exposure to, infectious pathogens (Fig. 6c and Supplementary Table 39).

## Discussion

Using data from multiple large cohort studies, our findings illustrate how a range of infections, namely influenza, herpes viruses, URTIs, LRTIs, skin and subcutaneous infections and a category of miscellaneous viral infections, are associated with increased regional brain atrophy and risk for future dementia. We extended these findings by identifying 35 immunologically relevant plasma proteins (among 942) that are differentially expressed after infection and associated with changes in brain regions vulnerable to infection-specific volume loss. We showed that a subset of these proteins was associated with cognitive decline and levels of AD and neurodegeneration plasma biomarkers, including PIK3CG, PACSIN2 and PRKCB. We also found genetic evidence linking infection-related proteins, such as ITGB6 and TLR5, with brain volume loss, suggesting that these proteins in particular may represent the molecular underpinnings linking infection with subsequent neurodegenerative disease (findings summarized in Fig. 6d).

The accelerated temporal lobe atrophy associated with infections in our study—although not robust to multiple-comparison correction—aligns with evidence of long-term reductions in this region resulting from immune insults, such as surgery and chemotherapy^27,28^. Although the absence of infection-related changes in other regions could be caused by a variety of factors, we speculate that these findings could be influenced by regional differences in brain-resident (for example, microglia) or infiltrating immune cells (for example, T cells), varying tropism of different pathogens across regions and cell types, and differing exposure to systemic inflammation via unique vasculature and blood–brain barrier conduits^29–34^. Each infection linked to brain volume loss in the BLSA was also associated with increased risk of incident dementia in the UK Biobank and/or the Finnish multicohort sample, in line with other studies^1–5^. Our results add to existing evidence by specifically implicating URTIs, as opposed to general respiratory infections or LRTIs^3,4^. Consistent with previous reports^2,4^, infections were associated with greatest risk of VaD, followed by all-cause and AD dementia, suggesting that postinfection immune alterations may be especially strong drivers of cerebrovascular pathology underlying large- and small-vessel diseases^35,36^. This hypothesis is supported by evidence that circulating inflammatory factors can impair the blood–brain barrier permeability, promote endothelial cell activation and induce neuroinflammation^37^.

The present study extends our understanding of how infections differentially relate to an immunological plasma proteome beyond select inflammatory markers. We suspect that the infection-related protein expression reported here reflect a pattern of long-term immune activation or systemic inflammation associated with prior infections, given that remote infections can alter the immune proteome through the training or priming of the immune system^12^. It is also possible that we are capturing the proteomic signature of individuals whose host immune traits render them susceptible to infection or maladaptive inflammatory responses^38^; this same host immune signature may influence one’s vulnerability to neurodegenerative processes. This hypothesis is supported by a recent study that linked predisposition to autoimmunity and AD risk^34^. A large number of the infection-related proteins that we detected have been previously associated with influenza, Zika and/or SARS-CoV-2 infection(s), but we did not find increases in C-reactive protein, TNF, IL-1β or IL-6, that is, proteins which are typically elevated as part of the acute response to infection. This finding is consistent with the idea that we are capturing the long-term immunoproteomic signature of past infections with an average interval between a first report of infection and blood draw for proteomic measurement of 14 years. Proteins most strongly associated with a participant’s infection status included peptides with validated antimicrobial activity (IFIT3 (ref. ^39^), DEFB4A^40^), products of AD risk genes (APOE^41^, TREM2 (ref. ^42^)) and several proteins that we recently showed to be associated with inflammatory dietary patterns and adverse neurocognitive outcomes (CDCP1 and CCL3 (ref. ^43^)). The comparatively large quantity of influenza-related proteins, many of which were strongly associated with other infections, may reflect infection-related alterations to the immune proteome that are insensitive to infectious etiology.

The present study identified plasma proteins increased after infection that were associated with brain atrophy (pathogenic), as well as proteins decreased after infection that were associated with preserved brain volume (protective). Our hypothesis that infections may increase levels of pathogenic immune proteins was supported and complemented by the finding that infection history, primarily influenza, was also associated with decreases in protective proteins, such as PIK3CG, PACSIN2 and PRKCB: proteins that have been previously implicated in neurodegeneration^44–46^, and were associated with reduced brain volume loss, preserved cognitive capacities and lower levels of AD pathology in our study. The pattern of infection-related downregulation of protective proteins could reflect the secondary consequences of attenuated host immunity induced by pathogens to evade immune detection^47^. Although the direction of protective and pathogenic effects of candidate proteins was generally consistent with respect to brain volumes, cognitive performance and biomarkers of AD and neurodegeneration, the unexpected associations of some candidate proteins (for example, protective proteins increased after infection) may reflect the temporally dynamic and pleiotropic relationship between inflammation and neurodegeneration that can vary dependent on disease stage^48,49^. Most candidate proteins that we identified were not highly expressed in CNS cells and only six showed associations with a biomarker of astrocyte-mediated neuroinflammation (GFAP), yet the consistent detection of their differing RNA (and even some protein) concentrations in AD brains suggests that these proteins emanating from peripheral immune cells may be especially important drivers of neurodegeneration.

Genetic variants modulating levels of ITGB6 and TLR5 were associated with brain volume loss across two independent cohorts, suggesting that these plasma proteins may be especially relevant to neurodegenerative mechanisms. ITGB6, the rate-limiting subunit of the αvβ6 integrin heterodimer, is elevated in the context of inflammatory stimuli (for example, infections and wound healing) where it is responsible for activation of the anti-inflammatory cytokine transforming growth factor-β1 and inhibiting immune cell infiltration, indicating that the pathogenic effects of increasing ITGB6 levels in the present study may be a sign of long-term, inadequately controlled systemic inflammation after infection^50,51^. TLR5 is a nominated therapeutic target by the AMP-AD consortium that is among the most conserved pattern recognition receptors, suggesting that its pathogenic effects on brain volumes may be attributed to chronic activation of its downstream proinflammatory pathways (for example, nuclear factor κ-light chain enhancer of activated B cells (NF-κB), TNF)^52^. As our analyses were largely limited to *trans*-pQTLs, distal regulation of ITGB6 and TLR5 through unknown intermediate genes may account for the ensuing effects on brain volume (for example, transcription factors near these *trans*-pQTLs that then influence expression of the plasma proteins). However, *trans*-pQTLs may also affect brain volume loss and protein levels through independent mechanisms, suggesting that the observed associations could also reflect pleiotropy. The discordant effects for some proteins across the BLSA and two-sample MR analyses indicate that the life-long contributions of genetic variants encoding candidate proteins on brain volumes captured by the ENIGMA consortium may not be adequately reflected in the observational window of the BLSA.

Although the present study has several strengths, including the use of longitudinal 3T MRI, a multicohort replication of dementia risk, state-of-the-art proteomics, and genetic techniques to support causality, it has several limitations. First, as evaluations at study visits were used to identify infections, inaccurate reporting or undiagnosed infections may have led to exposure misclassifications, a bias that would probably favor null results. Second, dementia analyses in external cohorts leveraged infection diagnoses in hospital discharge records, probably reflecting more severe cases compared with those reported in the BLSA, and with a 1-year time lag, probably captured the mixed effects of infections on dementia risk and increased infection vulnerability among individuals with prodromal or early dementia. Third, because of the retrospective nature of our study, we were unable to reliably control for the time between a participant’s infection and baseline MR scan. Although the levels of many inflammatory proteins—and plasma proteins more broadly—remain relatively stable across time^53,54^, the mean 14-year gap between infection documentation and blood collection in our study suggests that the infection-related proteomic signatures we identified may differ greatly from those seen in the acute or early post-acute phase of an infection. As a result of the exploratory nature of the present study, we did not adjust *P* values for multiple comparisons in our primary analysis of infection-related protein changes. Therefore, a fourth limitation is our study’s liberal threshold (uncorrected *P* < 0.05) and the lack of statistical significance for some of the results after FDR correction. Considering the extensive size of the proteomic panel, the limited number of cases for specific infections probably reduced the power to detect significant effects at an FDR-corrected threshold. Although we demonstrated that a large proportion of our infection-associated proteins has been externally replicated, future studies will be needed to validate associations between past infections and the expression of specific immune proteins. Fifth, as our study samples were predominantly of European ancestry, our findings may not be as generalizable to individuals of non-European ancestries. Last, although follow-up times for cases and controls did not differ for the six infections of interest, the attrition rate caused by death was elevated among those with a history of influenza and LRTIs. Although it is unclear how differential attrition may have affected our results, increased study dropout among those with a history of certain infections would probably bias estimates toward the null. Despite these limitations, our results show a link between infections, accelerated brain atrophy and increased dementia risk, while highlighting the immunological proteins by which infections may contribute to neurodegeneration.

## Methods

### Study sample

The present study used data from the BLSA, an ongoing longitudinal study designed to assess physical and cognitive measures in a cohort of community-dwelling volunteers^55^. Participants received comprehensive health and functional screening evaluations at each study visit (including ICD-9 documentation, blood draws and cognitive and physical examinations), which were completed by licensed healthcare professionals (for example, nurse practitioner, medical doctor). Study visits occurred biennially until 2005, then every 1–4 years depending on age (age <60 years, every 4 years; age 60–79 years, every 2 years; age ≥80 years, every year). For a subset of participants enrolled in the BLSA neuroimaging substudy, study visits occurred annually beginning in 1994. As a result of the BLSA’s continuous enrollment, participants entered the study at different times and thus varied with respect to follow-up times. Participants were selected if they had ICD-9 codes and MRI, SomaScan v.4.1 proteomic, Simoa biomarker, cognitive task, or GWAS data, as well as no neurological conditions that could affect brain structure or function (for example, strokes, seizures), and did not exhibit cognitive impairment at baseline or follow-up (that is, dementia, mild cognitive impairment (MCI), impaired but no MCI; procedures for determining cognitive status are detailed elsewhere^56^).

### Infection diagnoses

Using ICD-9 codes documented at each study visit beginning as early as 1958, along with infection-related medical codes derived from a recent study using the Danish National Patient Registry^1^, participants were categorized according to the presence (+) or absence (−) of an infection (Supplementary Table 1). ICD-9 codes in the BLSA were derived from one or more potential sources of information collected at enrollment and each study visit, including medical history records, comprehensive health and functional screening evaluations, and laboratory test results. Evidence of infections was adjudicated by a central panel of board-certified clinicians to ensure diagnostic reliability and accuracy. Infection categories with limited numbers of participants (<20 cases; for example, HIV, septicemia) were not considered in the current analyses owing to limited statistical power. Such classification parallels methodology used in recent studies, whereby the diagnosed sample primarily represents infected participants with clinical symptoms severe enough to report during follow-up patient healthcare consultations^4,8^. Participants with infection diagnoses documented at or before study enrollment or during follow-up visits (that is, a participant’s infection occurred in between study visits) were considered exposed. For positive and negative cases, the baseline visit for each participant was the earliest visit at which the presence or absence of an infection diagnosis was documented, respectively.

### 3T brain MRI

Beginning in 2009–2010, T1-weighted, magnetization-prepared, rapid gradient echo (MPRAGE) scans were acquired on a 3-T Philips Achieva (repetition time = 6.8 ms, echo time = 3.2 ms, flip angle = 8°, image matrix = 256 × 256, 170 slices, pixel size = 1 × 1 mm^2^, slice thickness = 1.2 mm). We applied a validated, Multi-atlas Region Segmentation Utilizing Ensembles (MUSE) anatomical labeling method specifically designed to achieve a consistent parcellation of brain anatomy in longitudinal MRI studies using T1-weighted sequences^57^. Analyses adjusted for intracranial volume (defined at age 70 years) and examined standardized values of total brain, gray matter, white matter and lobar volumes (frontal, parietal, occipital, temporal), as well as an AD-signature region volume (that is, the combined volume of hippocampus, parahippocampal gyrus, entorhinal cortex, posterior cingulate gyrus, precuneus and cuneus^21,22^). If an infection was significantly associated with a primary region of interest, we performed secondary analyses on lobar white/gray matter volumes. Standardized volumes of all 48 MUSE-labeled regions were used to explore change-related pQTLs (see below for further description of pQTL analyses).

### Immune plasma proteomics

Proteins were measured with the SOMAmer-based array method (SomaLogic)^58^, with analyses restricted to the Inflammation and Immune Response Panel which initially contained 938 SOMAmer reagents from the larger SomaScan v.4.1 (Supplementary Table 10). Plasma used for analyses was collected at baseline MR scan using standardized protocols and frozen at −80 °C until analysis; a subset of samples was collected at the time of a first positron emission tomography (PET) scan as part of a separate study. Samples from participants that did not pass SomaScan quality control (QC) criteria were excluded (*n* = 7). An additional two aptamers were included in analyses to capture variation in APOE3 and APOE4 and an additional four aptamers to capture variation in two proteins (CDCP1 and ITGA11) that we recently showed to be associated with inflammatory dietary and cognitive impairment^43^. Using 102 blind duplicates, aptamers with intra-assay coefficients of variation (CVs) > 50% were excluded (*n* = 2). The median intra-assay CV for aptamers on the Inflammation and Immune Response Panel was 4.5%. The median intra-assay CV for the aptamers measuring the 35 candidate proteins was 7%. Values were log_2_(transformed) and those beyond 5 s.d. values were winsorized. EntrezGene IDs were used as labels for corresponding aptamers.

### Cognitive assessment

Five cognitive composite scores across five domains—visuospatial ability, verbal memory, verbal fluency, executive functioning and attention—were calculated from standardized (converted to a *z*-score using the baseline mean and s.d.) and averaged individual task components^8,59^. Cognitive performance was measured at baseline and follow-up visits. As certain cognitive tasks were initiated in the BLSA at different periods according to protocol changes, composite scores for participants at each visit were computed from those tasks available at the time of assessment. Visuospatial ability was assessed using a modified version of the Educational Testing Service Card Rotations Test and two Clock Drawing Tests (CDTs), where participants were asked to draw the hands and face of clocks indicating 3:25 and 11:10. In the present study, a composite score was calculated using the average of the standardized *z*-scores from the Card Rotations Test and the mean of the CDTs. Verbal memory was assessed using immediate (sum of five learning trials) and long-delay free recall from the California Verbal Learning Test. Verbal fluency was calculated using Verbal Fluency Letters (F, A and S) and Verbal Fluency Categories (fruit, animals and vegetables). Executive function was assessed using Trail Making Test Part B and the Digit Span Backward subset of the Wechsler Adult Intelligence Scale (WAIS), revised. Attention was evaluated using Trail Making Test Part A and the Digit Span Forward subset of the WAIS, revised. Scores of Trail Making Test Parts A and B were first natural log(transformed), *z*-scored, and then signs inverted so that higher scores reflected higher performance, consistent with the direction of performance across other cognitive tasks.

### AD and neurodegeneration biomarkers

Aβ_40_, Aβ_42_, GFAP, NfL, and pTau-181 concentrations were measured using the Single Molecule Array (Simoa) Neurology 4-Plex E (N4PE) and pTau-181 (v.2) assays on the Simoa HD-X instrument (Quanterix). Plasma used for analyses was collected at baseline. Assays were run in duplicate and values averaged. CVs were 2.8%, 1.9%, 5.0%, 5.1%, and 4.4% for Aβ_40_, Aβ_42_, GFAP, NfL, and pTau-181, respectively. The Aβ_42/40_ ratio was used in analyses. Values for GFAP, NfL, and pTau-181 were log_2_(transformed) to correct for skewedness. Biomarker values were standardized and at a threshold of 5 s.d. values no outliers were detected.

### GWA, pQTLs and MRI

The pQTLs were obtained from the deCODE Genetics GWAS of SomaScan plasma protein levels^24^. For our analyses we incorporated pQTLs that met a genome-wide significant *P* value of 1.8 × 10^−9^. Genome-wide genotyping in the BLSA was performed using the Illumina 550K or NeuroChip platforms using standard QC procedures described previously^60^. Variants were excluded for poor call rate (missing >1%), violations of the Hardy–Weinberg equilibrium (*P* < 1 × 10^−6^), and limited minor allele frequency (<1%). Samples were excluded for poor genotyping efficiency (missing >2%), sex inconsistencies, cryptic relatedness (pihat >0.25), or if they were not of European ancestry by self-report or principal component detection. Imputation was performed for Illumina 550K or NeuroChip datasets separately with the Michigan Imputation Server MaCH (https://imputationserver.sph.umich.edu) using the HRC r1.1.2016 reference panel. After imputation, datasets were merged, overlapping samples were removed, and SNPs with low imputation quality (*R*^2^ < 0.9), minor allele frequencies <1%, Hardy–Weinberg equilibrium *P* values < 1 × 10^−6^, and those that did not overlap between the two datasets (missingness <99%) were excluded. The final genetic dataset included 5,439,477 SNPs and 1,184 individuals; of them, 469 had neuroimaging data available in the present study. BLSA data were not included in the ENIGMA consortium, which was used to validate findings with two-sample MRI.

For two-sample MRI, we used deCODE genome-wide significant plasma pQTLs as genetic instruments for candidate plasma proteins. The outcome (brain atrophy) was characterized using summary statistics from a GWAS of longitudinal MRI-derived total brain volumes from the ENIGMA consortium (*n* = 15,640)^26^. Plasma pQTLs were pruned to remove genetic variants in linkage disequilibrium (LD) (*r*^2^ < 0.05) as previously described^61^ with 1000 Genomes Project European as the reference panel. If the allele information for a given pQTL was not reported, the LDproxy Tool (https://ldlink.nih.gov/?tab=ldproxy) was used to identify another SNP (*r*^2^ > 0.80) and its allele information for replacement; if multiple replacement SNPs for a given pQTL were identified, we used the SNP with the highest *r*^2^ value^62^. The random-effect, inverse variance-weighted estimate (for proteins with multiple genetic instruments) or Wald’s ratio estimate (for proteins with a single genetic instrumental variable) was used for primary analyses. Sensitivity analyses were performed to assess the violation of MR assumptions using MR-Egger, weighted-median and weighted modes. Among candidate proteins with sufficient numbers of available instruments, heterogeneity between causal estimates was tested using Cochran’s *Q* and/or the Mendelian Randomization Pleiotropy Residual Sum and Outlier (MR-Presso) Global test, and horizontal pleiotropy assumptions were tested using the MR-Egger intercept.

### Covariates

For BLSA analyses, baseline age (years), sex (male/female), race (white/non-white), and education level (years) were defined based on participant reports. Non-white race includes Black, American Indian or Alaska Native, Chinese, Filipino, Hawaiian, Japanese, other Asian or Pacific Islander, and other non-white. *APOE*ε4 carrier status (0 ε4 alleles/≥1 ε4 alleles/missing) was defined via PCR with restriction isotyping using the type IIP enzyme Hhai or the Taqman method. Estimated glomerular filtration rate (eGFR)-creatinine was defined from blood samples using the CKD-EPI (Chronic Kidney Disease Epidemiology Collaboration) criteria. Comorbid diseases that represent potential confounders were defined using a comorbidity index calculated as the sum (score range: 0–8; converted to a percentage to account for missing data) of eight conditions: obesity, hypertension, diabetes, cancer, ischemic heart disease, chronic heart failure, chronic kidney disease, and chronic obstructive pulmonary disease^63^. A BMI ≥ 30 kg m^−^^2^ and a glycated hemoglobin (HbA1c) ≥ 6.5% (48 mmol mol^−1^) were used to define obesity and diabetes, respectively; comorbid conditions were identified by the study physician at each visit from medical history.

### Protein characterization

Several analytical tools were used to understand the biological implications of candidate proteins. A complete description is provided in Supplementary Methods.

### External cohorts

A complete description of external cohorts (that is, UK Biobank, Finnish multicohort sample, the GenS study and the ARIC study) is provided in Supplementary Methods.

### Ethics statement

The BLSA protocol was approved by the Institutional Review Board of the National Institute of Environmental Health Science, National Institutes of Health (NIH, protocol no. 03AG0325). All participants gave written informed consent before participation and deidentified data were used for analyses. BLSA participants were not financially compensated. The UK Biobank study was approved by the National Health Service National Research Ethics Service (protocol no. 11/NW/0382). All participants gave informed consent. The FPS study was approved by the ethics committee of the Hospital District of Helsinki and Uusimaa (protocol no. HUS/1210/2016), the HeSSup study was approved by the ethics committee of Turku University Hospital and the Finnish Population Register Centre (protocol no. VRK 2605/410/14), and the STW study was approved by the ethics committee of the Finnish Institute of Occupational Health. All participants gave informed consent. In the FPS study, there were additional participants from whom only deidentified register data were collected and thus no consent was required. The ARIC study protocols were approved by the institutional review boards at each participating center: University of North Carolina at Chapel Hill, Chapel Hill, NC; Wake Forest University, Winston-Salem, NC; Johns Hopkins University, Baltimore, MD; University of Minnesota, Minneapolis, MN; and University of Mississippi Medical Center, Jackson, MS. All ARIC study participants gave written informed consent at each study visit; proxies provided consent for participants who were judged to lack capacity.

### Statistics and reproducibility

Statistical significance was defined at two-sided *P* < 0.05. Analyses were performed using R (v.4.2.2) and Stata/MP (v.17). R packages included nlme (v.3.1.162), data.table (v.1.15), tidyverse (v.2.0), ggplot2 (v.3.5.1), TwoSampleMR (v.0.5.6), MendelianRandomization (v.0.6.0) and MR-Presso (v.1.0). No statistical methods were used to predetermine sample sizes, but they were similar to those reported in previous publications^2,8^. Data distributions were visually inspected to confirm assumptions for statistical tests (for example, normality and equal variance), but this was not formally tested. Randomization of participants was not necessary and therefore not employed. Investigators were blinded during data collection and throughout analyses. Graphs were generated in R, Graphpad Prism (v.9.3.1) and the Biorender platform (https://www.biorender.com).

### BLSA

In the BLSA, linear mixed-effect models were used to examine associations of infections with longitudinal rates of change in brain volumes. In addition to adjusting for intracranial volume (defined at age 70 years), models included the following covariates: baseline age, sex, race, education, *APOE*ε4, comorbidity index, and the interactions of age, sex, race, education, *APOE*ε4, and comorbidity index with time. Random effects of intercept and time with unstructured covariance were included to account for the within-subject correlation of the repeated assessments. In addition to comparing participants with and without a history of a specific infection (for example, influenza versus noninfluenza; primary analysis), sensitivity analyses compared participants with history of a specific infection with those without a history of any infection (for example, influenza versus no history of any infection). Multiple linear regression models adjusting for baseline age, sex, race, education, *APOE*ε4, comorbidity index, and eGFR-creatinine were used to examine associations of infections with SomaScan Inflammation and Immune Response panel proteins. Sensitivity analyses were performed to adjust for a participant’s total concurrent frequency of infections observed at the time of infection diagnosis. Similar linear mixed-effect models were used to examine associations of candidate proteins with longitudinal rates of change in brain volumes and cognitive performance. Multiple linear regression models, similar to those described above, were used to examine the associations of candidate proteins with AD and neurodegeneration biomarkers. Separate, linear mixed-effect models incorporated two-way and three-way interaction terms (infection × protein, infection × protein × time) to examine whether an infection diagnosis modified the association of immunological proteins with longitudinal brain volume change. Multiple linear regression models (adjusted for covariates used in the pQTL discovery cohort^24^, namely baseline age and sex) were used to examine associations of pQTLs with SomaScan protein levels in BLSA participants, and linear mixed-effect models (adjusted for the aforementioned BLSA covariates) were used to examine the associations of pQTLs with longitudinal rates of change in brain volumes in the same participants. Welch’s *t*-test was used to compare expression levels of genes encoding candidate proteins (cognate genes) between AD and control participants across neurovascular cell types^64^.

### UK Biobank and Finnish multicohort sample

Using Cox proportional hazards models, hazard ratios and 95% CIs were computed for the associations of infections with all-cause dementia, AD dementia, and VaD^2,65^. Follow-up for dementia commenced at study entry and continued until the first record of dementia, death, loss to follow-up, or end of the hospital records, whichever occurred first. For each group of infections, participants with a record of a relevant infection on or before study entry were considered exposed from the start of the follow-up. Those with their first record of a relevant infection during follow-up were considered nonexposed until the infection and exposed thereafter. Those who had no record of a relevant infection before the end of the follow-up were considered unexposed across the entire follow-up period. In the UK Biobank: model 1 was adjusted for age (using time since birth as the time scale), sex, and socioeconomic status; model 2 was additionally adjusted for BMI, hypertension, diabetes, and *APOE* genotype; and model 3 had further adjustments for alcohol consumption and smoking. In the Finnish multicohort sample, model 1 was adjusted for age (using time since birth as the time scale), sex, and socioeconomic status, and model 2 was additionally adjusted for hypertension and diabetes. Both models also took the within-cohort clustering of participants into account using cohort-specific baseline hazards. Ascertainment bias can occur if the medical attention received for an infection increases the probability that an underlying dementia is noticed and recorded, whereas reverse causation can occur if systemic changes related to the long preclinical phase of dementia increase susceptibility to infections^2,66,67^. To reduce the risk of these biases, we additionally conducted 1-, 5- and 10-year lag analyses, in which we excluded the first 1, 5 and 10 years of follow-up after infection, respectively. P.N.S. had full access to these data and took responsibility for their integrity and the data analysis.

### GenS

Standardized β coefficients for each protein–cognitive domain relationship were derived from linear mixed-effect models that corrected for relatedness between individuals (that is, kinship matrix) and adjusted for age, sex, depression diagnosis, clinic study site, and sample storage time.

### ARIC

Dementia risk was assessed using binary logistic regression models adjusted for age, sex, race center, education, *APOE*ε4, eGFR-creatinine, and cardiovascular risk factors (BMI, diabetes, hypertension, and current smoking status). Associations with AD and related dementia (ADRD) biomarkers were assessed using multiple linear regression models adjusting for similar covariates.

### Reporting summary

Further information on research design is available in the Nature Portfolio Reporting Summary linked to this article.

## Supplementary information

Supplementary Information, Methods, Figs.1–13 and References.

Reporting Summary

Supplementary Tables 1–39.

## Data Availability

All data generated in the present study are included in this article (and Extended Data Figs. 1–8, Supplementary Figs. 1–13 and Supplementary Tables 1–39), available on reasonable request, or in an online public repository. Participants did not consent to unrestricted data sharing at the time of the study conducted for BLSA. Researchers are welcome and encouraged to request use of BLSA data for scientific projects. Anonymized data not published within this article may be shared upon request from qualified investigators for purposes of replicating procedures and findings. Researchers who wish to use BLSA data are encouraged to develop a pre-analysis plan that can be submitted for approval (https://blsa.nia.nih.gov/how-apply). BLSA proteomic data are publicly available via the Alzheimer’s Disease Data Initiative as part of the participation in the Global Neurodegeneration Proteomics Consortium (https://www.neuroproteome.org). To apply and request access to BLSA proteomics data for purposes of reproducibility, researchers should submit a pre-analysis plan for approval (https://blsa.nia.nih.gov/how-apply). Data, protocols and other metadata of the UK Biobank are available to the scientific community upon request in accordance with the UK Biobank data-sharing policy (https://www.ukbiobank.ac.uk/enable-your-research/apply-for-access). In the Finnish cohort studies, linked health records require separate permission from the Finnish Institute of Health and Welfare and Statistics Finland (https://thl.fi/en/web/thlfi-en/statistics-and-data/data-and-services/research-use-and-data-permits; https://tilastokeskus.fi/tup/mikroaineistot/aineistojen-yhdistaminen_en.html). ARIC proteomic data are available through the NHLBI Biologic Specimen and Data Repository Information Coordinating Center (https://biolincc.nhlbi.nih.gov/studies/aric). Additional requests for clinical or proteomic data from individual investigators may be submitted to the ARIC steering committees and will be reviewed to ensure that data can be shared without compromising patient confidentiality or breaching intellectual property restrictions. Participant-level demographic, clinical and proteomic data may be partially restricted based on previously obtained participant consent. Data-sharing restrictions may also be applied to ensure consistency with confidentiality or privacy laws and considerations (https://sites.cscc.unc.edu/aric).

## Code availability

The statistical code programs are publicly available through GitHub at https://github.com/dugganmr and https://github.com/pyrysipila.
