# Supplementary material for: Proteomics identifies potential immunological drivers of postinfection brain atrophy and cognitive decline
Source: Nat Aging. Author manuscript; Available in PMC 2024 Oct 11. (PMC11408246; doi:10.1038/s43587-024-00682-4)
Supplement: Supp information 1 [file NIHMS2021124-supplement-Supp_information_1.pdf]

nature aging

Supplementary information https://doi.org/10.1038/s43587-024-00682-4

Proteomics identifies potential immunological drivers of postinfection brain atrophy and cognitive decline

In the format provided by the authors and unedited

Supplementary methods
External cohorts
UK Biobank
The UK Biobank is a prospective cohort study. Baseline assessments were conducted at 22 assessment centers in England, Scotland, and Wales from 13 March 2006 to 1 October 2010. Participants without a record of hospitalisation due to dementia at baseline were eligible for analysis. In the UK Biobank, exposure to infections was ascertained from primary and secondary diagnoses in linked hospital discharge records from HES APC (Hospital Episode Statistics–Admitted Patient Care) (for England), SMR01 (Scottish Morbidity Records–General/Acute Inpatient and Day Case Admissions), and PEDW (Patient Episode Database for Wales) with ICD10 medical codes corresponding to the ICD9 codes used in BLSA. Dementia outcomes were ascertained from linked hospital discharge records and death certificates using ICD10 codes F00-F03, F05.1, G30, G30.0, G31.1, and G31.8 for all-cause dementia, F00 and G30 for Alzheimer's disease, and F01 for vascular dementia, and ICD9 codes 290.0-290.4, 290.8, 290.9, 294.1, 294.2, 331.0, 331.1, 331.2, and 331.8 for all-cause dementia, 331.0 for Alzheimer's disease, and 290.4 for vascular dementia (there were no ICD-8 codes in the data). Records were available for the UK Biobank until 23 March 2021 in England and Scotland and 28 February 2018 in Wales. Covariates included sex, socioeconomic status (low, intermediate, high), body mass index (<18.5, 18.5-24.9, 25.0-29.9, ≥30 kg/m^2), hypertension (yes, no), diabetes mellitus yes, no), alcohol consumption (never, former, 3 classes of moderate, intermediate, heavy), and smoking (never, former, current). Socioeconomic status was based on self-reported educational attainment: low (no qualification), high (college or university degree), or intermediate (all others). Body mass index was based on measured height and weight. Hypertension was defined as the mean of two systolic and diastolic blood pressure measurements ≥140/90mmHg, self-reported hypertension, or self-report for antihypertensive medication. Blood pressure was measured by an automated Omron device, or manually if automated reading was not available. Diabetes mellitus was defined as glycated haemoglobin ≥48 mmol/mol (6.5%), self-reported diabetes, or self-report for antidiabetic medication (insulin). Glycated haemoglobin was measured by HPLC analysis on a Bio-Rad VARIANT II Turbo. *APOE*ε4 carrier status was based on two single-nucleotide polymorphisms, rs429358 and rs7412, that were directly genotyped using UK Biobank Axiom array. Based on self-reported frequency of alcohol drinking and average monthly and weekly consumption of different alcoholic beverages, participants were classified as never-drinkers, ex-drinkers, those drinking on special occasions only, those drinking 1-3 times/month, weekly moderate drinkers (women consuming 1–14 drinks per week and men consuming 1–21 drinks per week), weekly intermediate drinkers (women consuming 15–20 drinks per week and men consuming 22–27 drinks per week), and weekly heavy drinkers (women consuming ≥21 drinks per week and men consuming ≥28 drinks per week) with one drink defined as 10g of pure alcohol. Smoking was self-reported.

Finnish multicohort sample
The Finnish Multicohort Sample comprised pooled and harmonized individual-participant data from all three Finnish prospective cohort studies that are part of the IPD-Work consortium: the Finnish Public Sector study (FPS), the Health and Social Support study (HeSSup), and the Still Working study (STW)[1]. Study entry was from 1 March 1986 to 1 January 2005. Participants who were at least 18 years old and without a record of dementia at baseline were eligible for analysis. Hospital inpatient discharge information from the Finnish Institute for Health and Welfare was used to ascertain exposure to infections with ICD10 medical codes corresponding to the ICD9 codes used in BLSA. Dementia outcomes were ascertained from inpatient hospital discharge records (from the Finnish Institute for Health and Welfare) and death certificates (from Statistics Finland), and additionally from hospital outpatient records (from the Finnish Institute for Health and Welfare) and medication reimbursement entitlements for the treatment of dementia (from Finnish Social Insurance Institution). Both primary and secondary

diagnoses were included. All-cause dementia comprised ICD10 codes F00-F03, F05.1, G30, G30.0, G31.1, and G31.8; Alzheimer's disease F00 and G30; and vascular dementia F01. The ICD9 codes were 290, 2900A, 2941A, 3310A, 3311A, 3312X, 3318X, and 4378A for all-cause dementia; 3310A for Alzheimer's disease; and 4378A for vascular dementia. The ICD8 codes were 29000, 29010, 29011, 29019, 34791, and 34792 for all-cause dementia and 29010 for Alzheimer's disease; there was no ICD8 codes for vascular dementia. Records for the Finnish Multicohort Sample were available until 31 December 2016 in FPS and STW and 31 December 2012 in HeSSup. Covariates included sex, socioeconomic status (low, intermediate, high), hypertension (yes, no), and diabetes mellitus (yes, no). Socioeconomic status was based on education recorded by Statistics Finland (FPS), self-reported education (HeSSup), or self-reported occupational grade (STW). Hypertension and diabetes mellitus were based on medication reimbursement entitlements.

Generation Scotland (GenS) study

We used previously published results from the Generation Scotland: The Scottish Family Health Study (GenS), a population-based cohort which recruited > 24,000 Scottish individuals between 2006 and 2011, including 1,065 participants with protein and cognitive measurements[2]. Each participant's clinical visit included a blood draw and detailed cognitive assessment. Proteins were measured using the SomaScan v4.0 assay. Cognition was measured across five domains. Verbal memory was computed as the sum of Immediate and delayed recall of one oral story from the Wechsler Logical Memory Test, where details correctly recalled about the story were recorded as points. Verbal Fluency was assessed using the Controlled Oral Word Association task (letter C, F, L), reflecting the number of words named with a 1 min. Processing speed was measured using the Wechsler Digit Symbol Substitution Task, specifically counts of correct pairs of digits recoded to symbols over 2 min. Non-verbal reasoning was assessed using the Matrix Reasoning test, which reflected the number of correct answers identifying missing elements in patterns presented as matrices. A measure of general cognition was computed as the first unrotated principal component combining logical memory, verbal fluency, processing speed and vocabulary (measured using the Mill Hill Vocabulary test). Participants with cognitive scores beyond 3.5 SDs were excluded from analyses.

Atherosclerosis Risk in Communities (ARIC) study

The Atherosclerosis Risk in Communities (ARIC) study is a prospective epidemiologic study conducted in four U.S. communities (Forsyth County, NC; Jackson, MS; the northwest suburbs of Minneapolis, MN; and Washington County, MD), which enrolled 15,792 white and black participants aged 45-64 between 1987-1989[3]. After initial enrollment, participants had four additional in-person visits: Visit 2 (1990-1992), Visit 3 (1993-1995), Visit 4 (1996-1999), Visit 5 (2011-2013). At Visit 5, blood was drawn for proteomic and plasma biomarker analysis, and dementia status was assessed. Proteins were measured using the SomaScan v4.0 assay; SomaLogic protein quality control steps in ARIC have been described in detail previously[4]. Using a set of 197 blind duplicates, the median CV for proteins included in this analysis was 6.3%. Protein levels were $\log_2$ transformed to correct for skewness. $A\beta_{40}$, $A\beta_{42}$, GFAP, NfL and pTau-181 concentrations were measured using the Single Molecule Array (Simoa) Neurology 4-Plex E (N4PE) and pTau-181 (V2) assays on the Simoa HD-X instrument (Quanterix). Using 90 blind duplicates, CVs were 7.3, 8.5, 2.3, 3.7 and 5.6% for $A\beta_{40}$, $A\beta_{42}$, GFAP, NfL and pTau-181, respectively. $A\beta_{42/40}$ ratio was used in analyses. Values for GFAP, NfL and pTau-181 were $\log_2$ transformed to correct for skewness. Biomarker values were standardized and those beyond 5 SDs were excluded. Dementia diagnosis was adjudicated through a surveillance approach using cognitive assessment tests, telephone screening, informant ratings, hospital records, and death record review, as previously described[5]. In brief, participants received a comprehensive cognitive exam and a functional assessment that included the Clinical Dementia Rating Scale (CDR) and Functional Activities Questionnaire (FAQ). Using this data, dementia was classified based

on the NIA/AA (National Institute on Aging and Alzheimer's Association) and the Diagnostic and Statistical Manual of Mental Disorder – Fifth Edition (DSM-5) criteria.

Protein characterization

Enriched biological pathways and upstream regulators were identified using Ingenuity Pathway Analysis (Qiagen Inc; version 01-22-01), a bioinformatics application that facilitates the analyses and interpretation of '-omics' data using manually curated content available through the Ingenuity Knowledge Base[6]. All candidate proteins were mapped to the Ingenuity Knowledge Base (i.e., each candidate protein was linked to a matching gene ID). Protein levels reflected differential expression associated with infection; duplicates (i.e., proteins differentially expressed with more than one infection) were resolved by consolidating the gene ID (e.g., Uniprot, Entrez) and using the maximum expression value of the two aptamers. Benjamini-Hochberg FDR adjusted *p*-values derived from Fisher's exact tests quantified the probability of overlap between candidate proteins and molecules (genes, transcripts, proteins, drugs) known to exist within a specific pathway or process due to random chance. Protein interaction networks were assessed using STRING (Search Tool for the Retrieval of Interacting Genes/Proteins) (https://string-db.org), a database and visualization tool that integrates publicly available sources of information to provide a comprehensive understanding of protein–protein interaction (PPI) networks. PPI *p*-values derived from an explicit null model to account for the non-uniform distribution of the connectivity degrees of network proteins (i.e., a random graph with given degree sequence model) reflected the likelihood of proteins having more interactions among themselves than what would be expected for a random set of proteins of the same size drawn from the genome, suggesting if the proteins are at least partially biologically connected as a group[7]. For comparative gene expression across 76 cell types, we used consensus transcript expression levels (normalized Transcripts per Million; nTPM) from the Human Protein Atlas (https://www.proteinatlas.org/about/). If a CNS cell (i.e., astrocytes, excitatory/inhibitory neurons, microglia, oligodendrocytes, oligodendrocyte precursor cells) was among the top 5 cell types with the highest expression of a given gene, it was considered highly expressed in a CNS cell; a gene was considered undetectable in CNS cells if all CNS cell types maintained ≤1 nTPM. Supplemental information relevant to AD, including expression levels (RNA, protein) in post-mortem brain tissue, was obtained from the AD Knowledge Portal (https://adknowledgeportal.synapse.org), a platform for accessing data, analyses, and tools generated by the Accelerating Medicines Partnership (AMP-AD) Program for AD and other NIA-supported programs. Expression levels in neurovasculature cell types were obtained from the Human BBB (https://twc-stanford.shinyapps.io/human_bbb/), a transcriptomic dataset generated using VINE (Vessel Isolation and Nuclei Extraction)-seq[8]. The Open Targets Platform (https://platform.opentargets.org) was used to identify medications that target specific proteins.

Supplementary figures

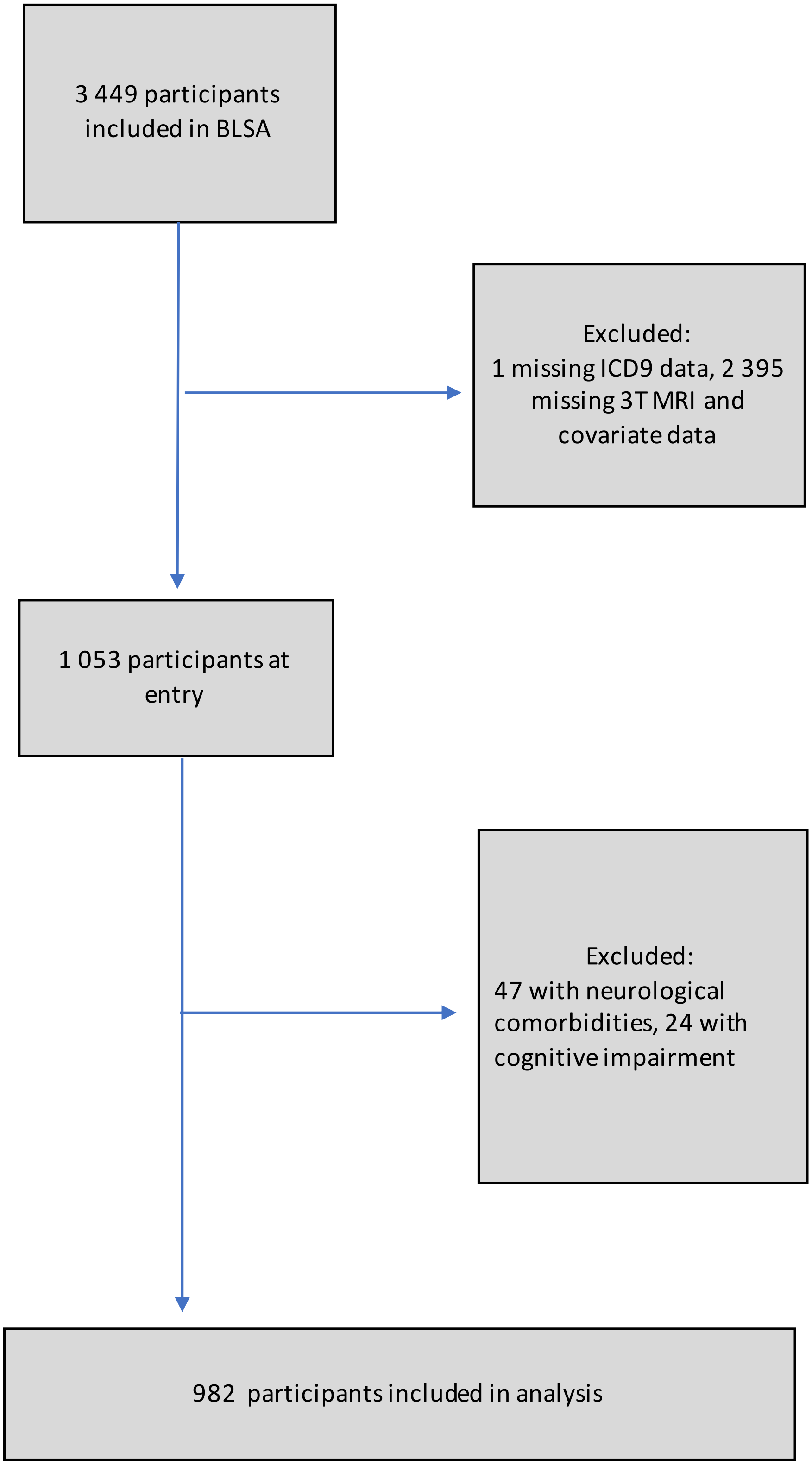

Supplementary Figure 1. Flow chart of participant selection for brain volume analyses in BLSA.

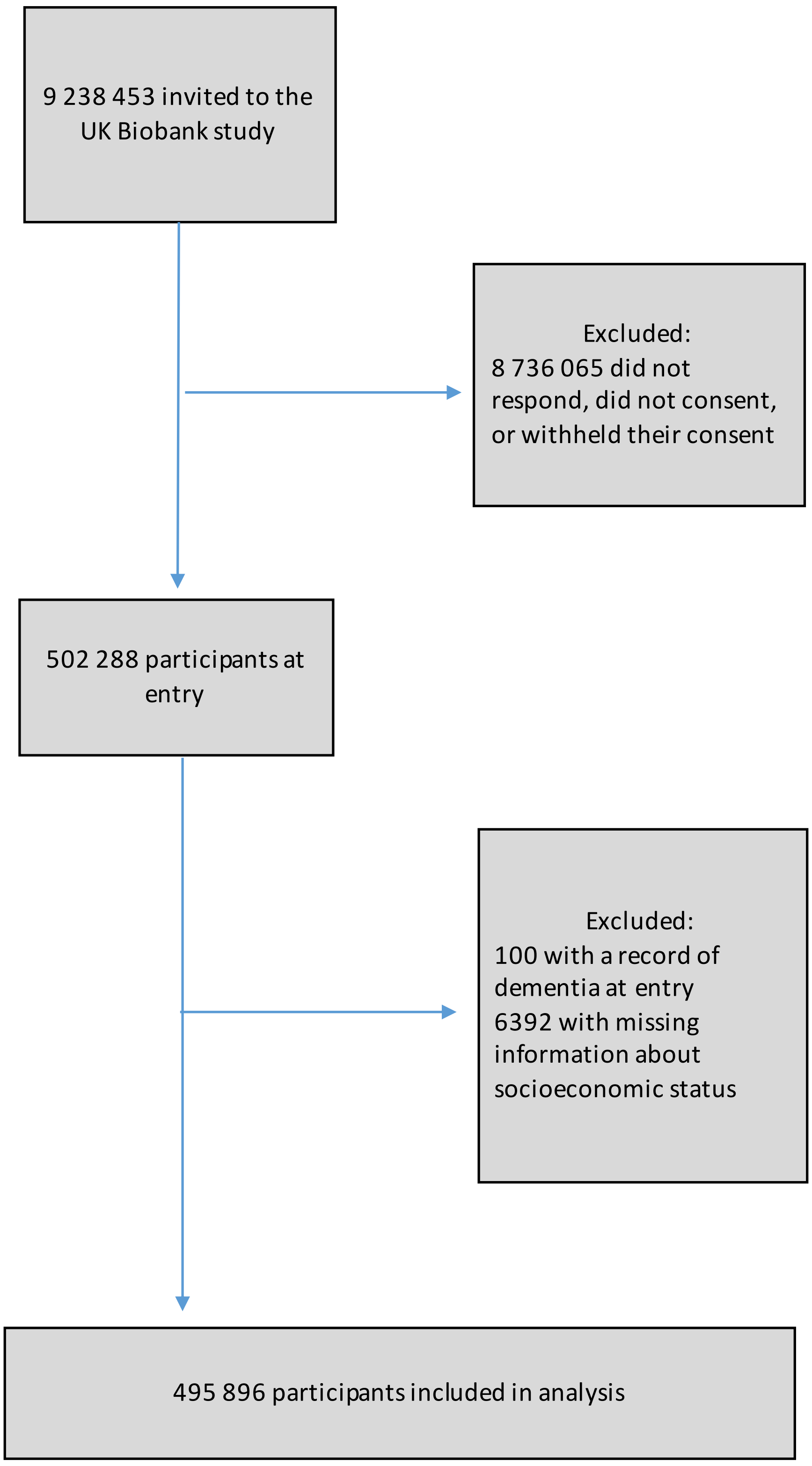

Supplementary Figure 2. Flow chart of participant selection in the UK Biobank.

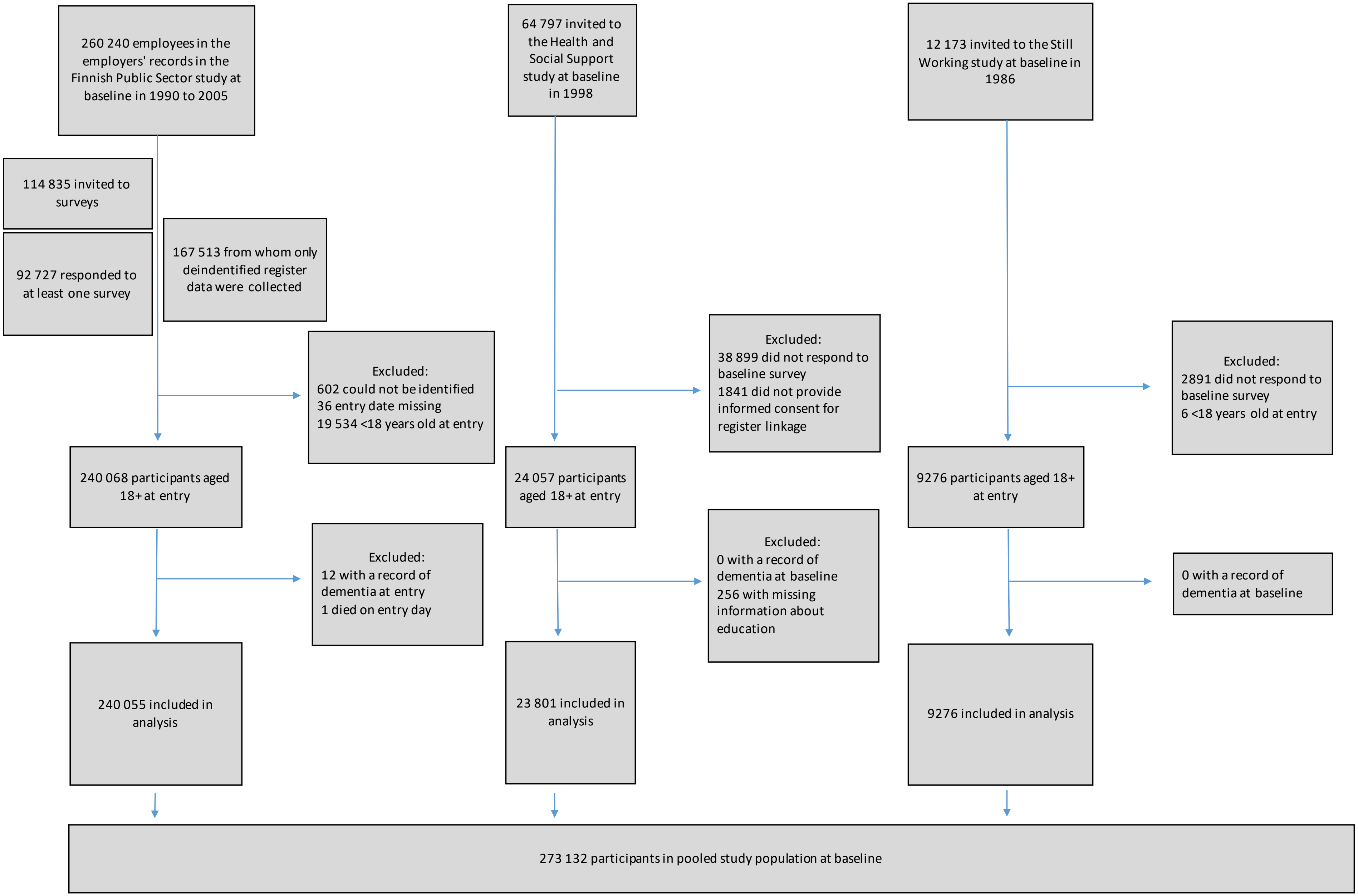

Supplementary Figure 3. Flow chart of participant selection in the Finnish multicohort sample, which consisted of the Finnish Public Sector study, the Health and Social Support study, and the Still Working study.

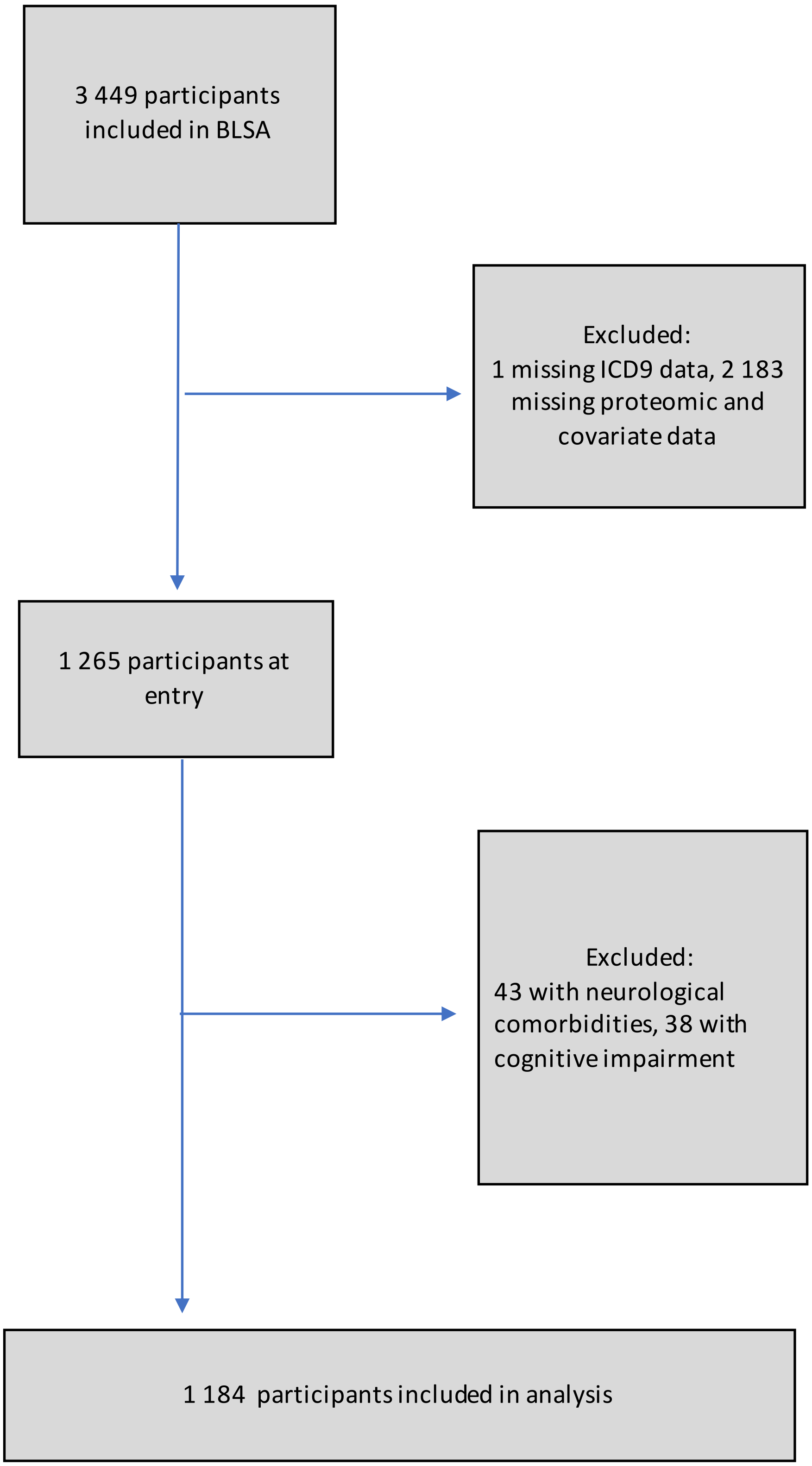

Supplementary Figure 4. Flow chart of participant selection for immune proteomics analyses in BLSA.

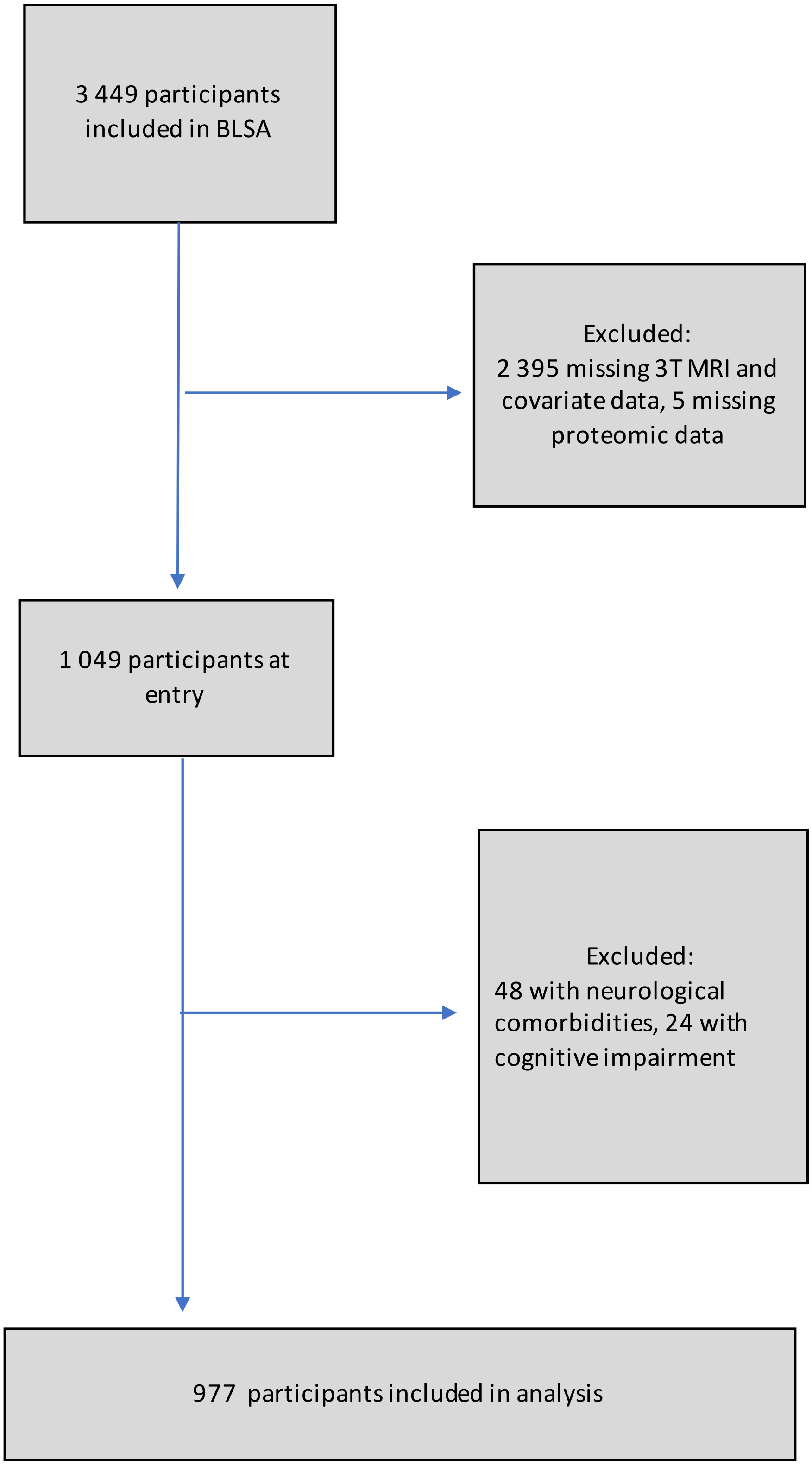

Supplementary Figure 5. Flow chart of participant selection for immune proteomics-MRI analyses in BLSA.

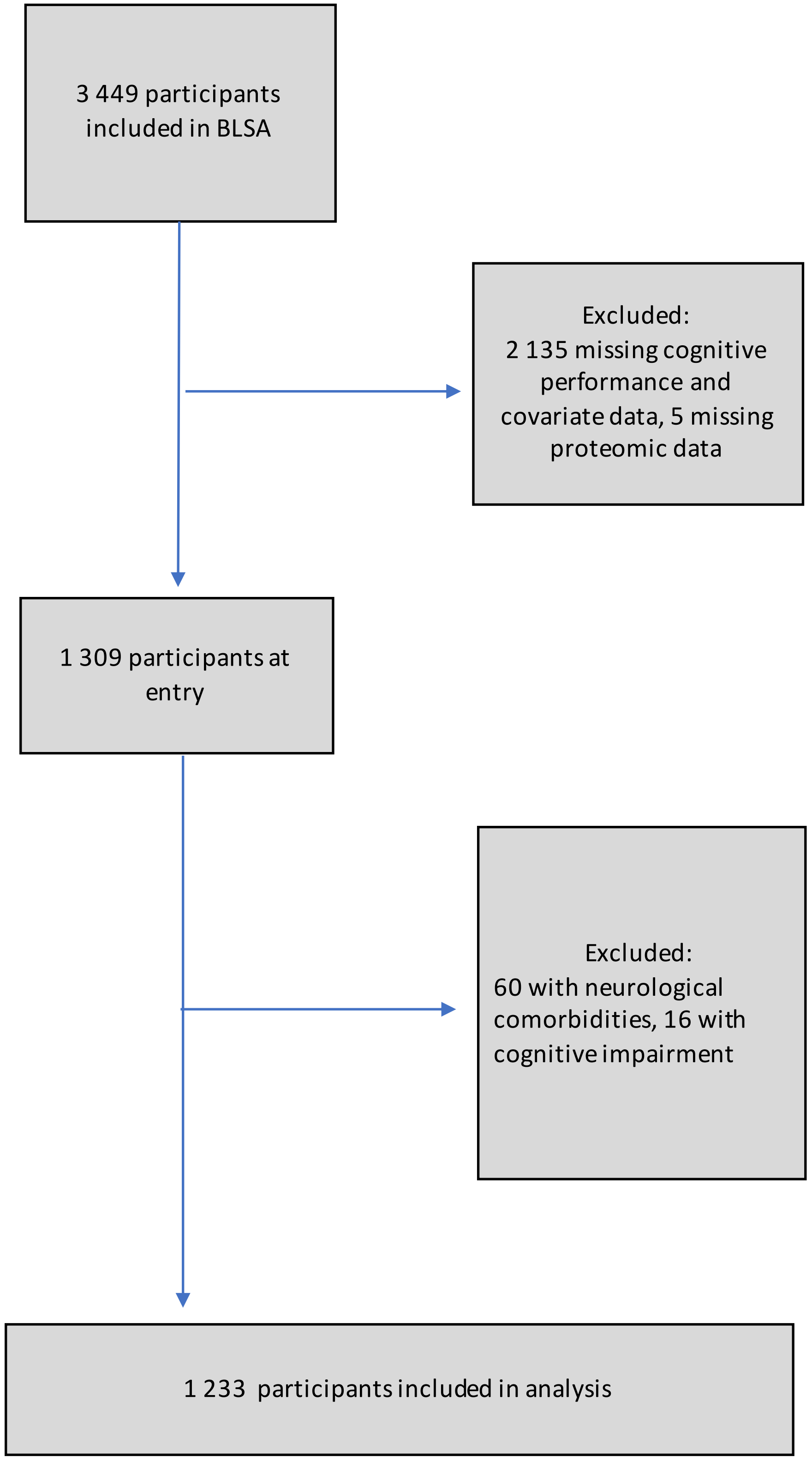

Supplementary Figure 6. Flow chart of participant selection for cognitive performance analyses in BLSA.

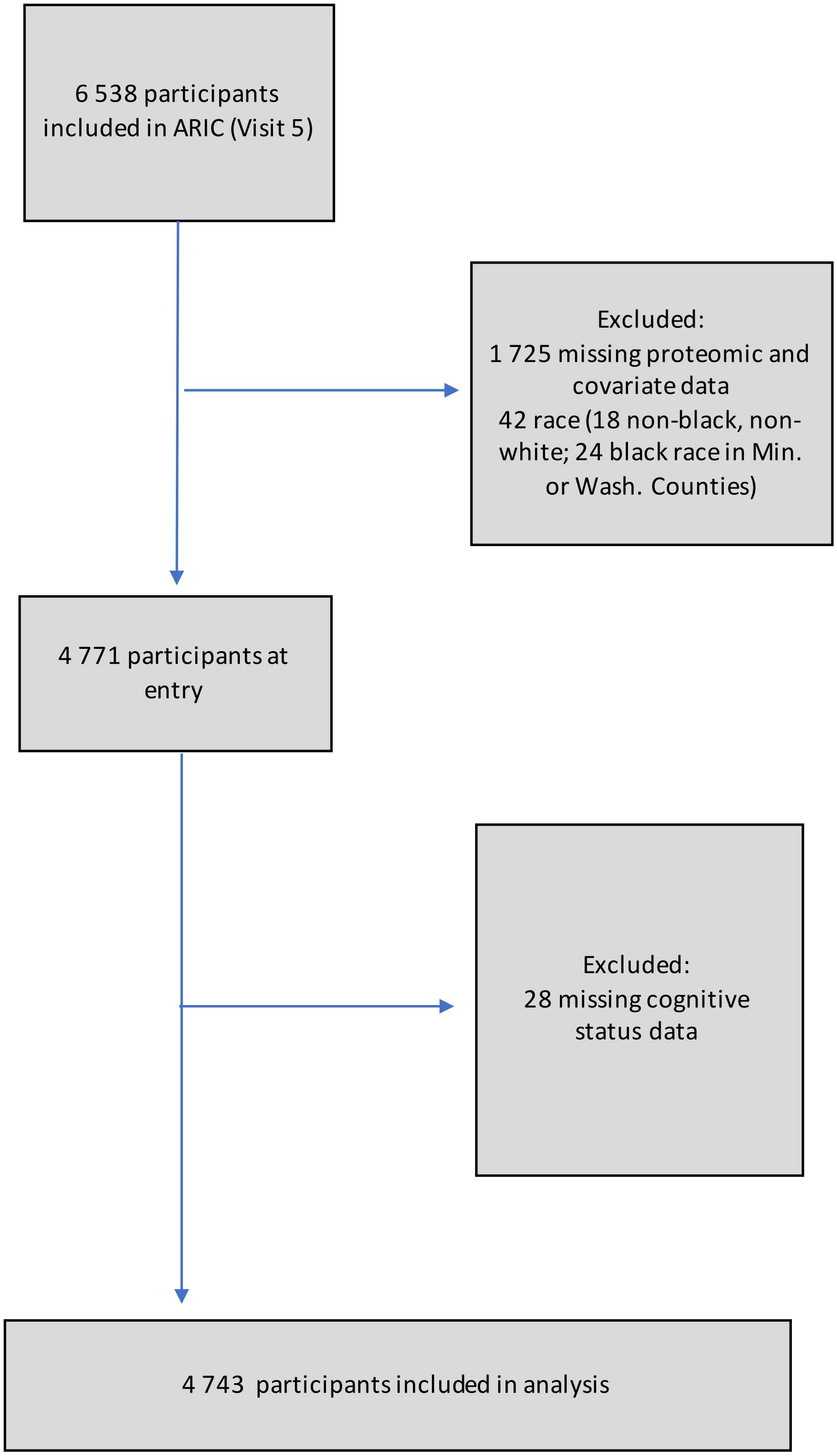

Supplementary Figure 7. Flow chart of participant selection for dementia risk analyses in ARIC.

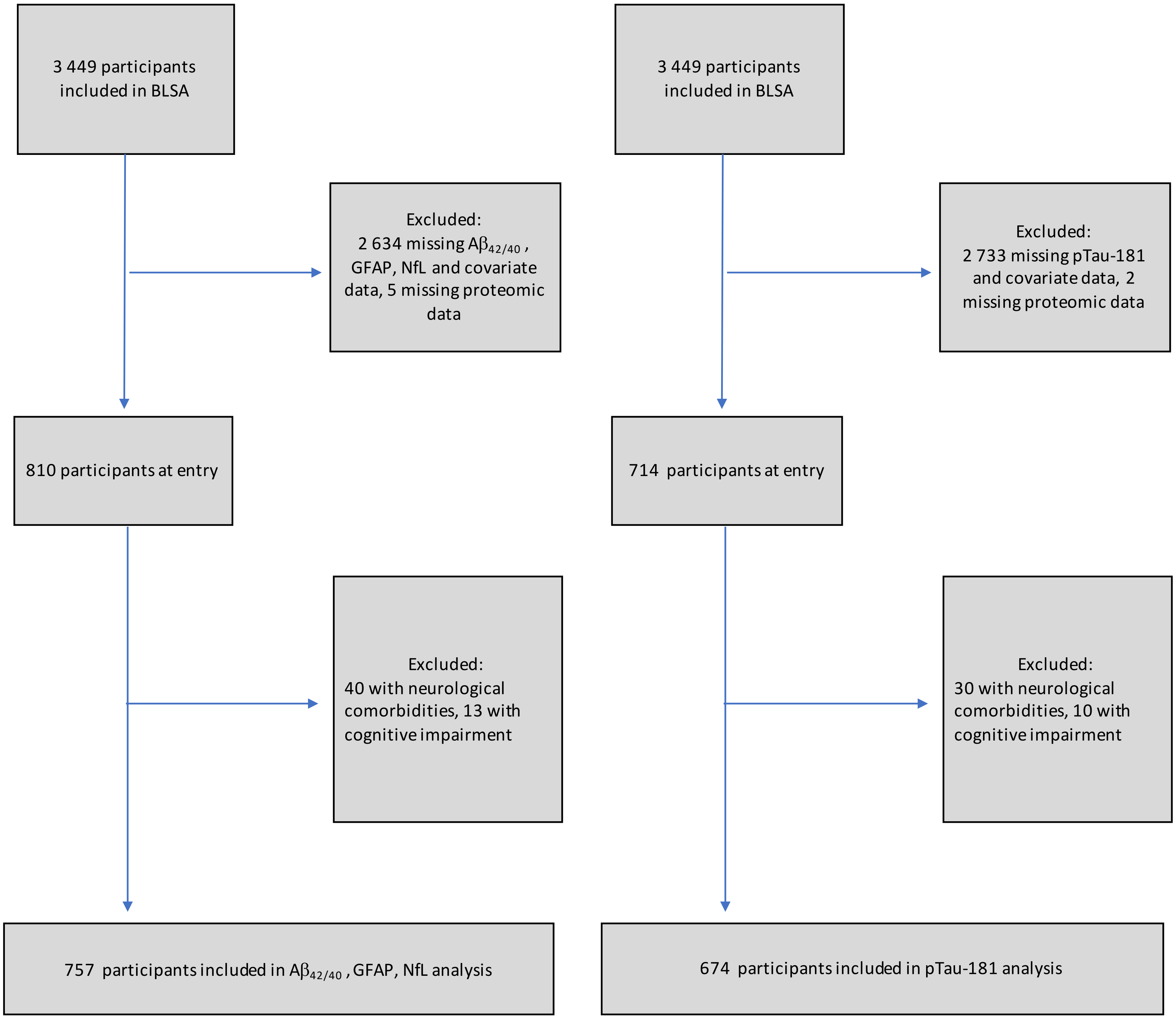

Supplementary Figure 8. Flow chart of participant selection for plasma ADRD biomarker analyses in BLSA.

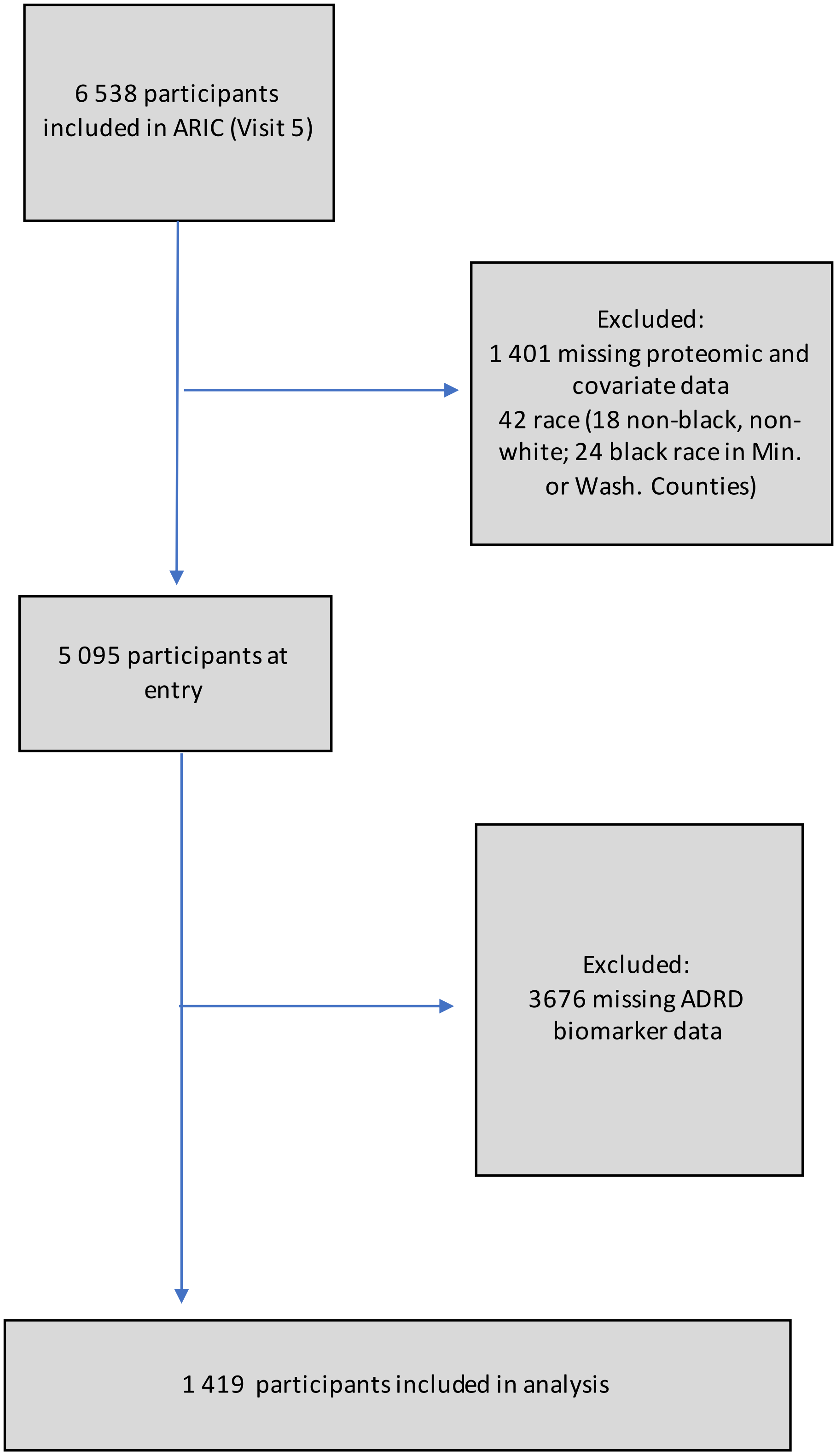

Supplementary Figure 9. Flow chart of participant selection for plasma ADRD biomarker analyses in ARIC

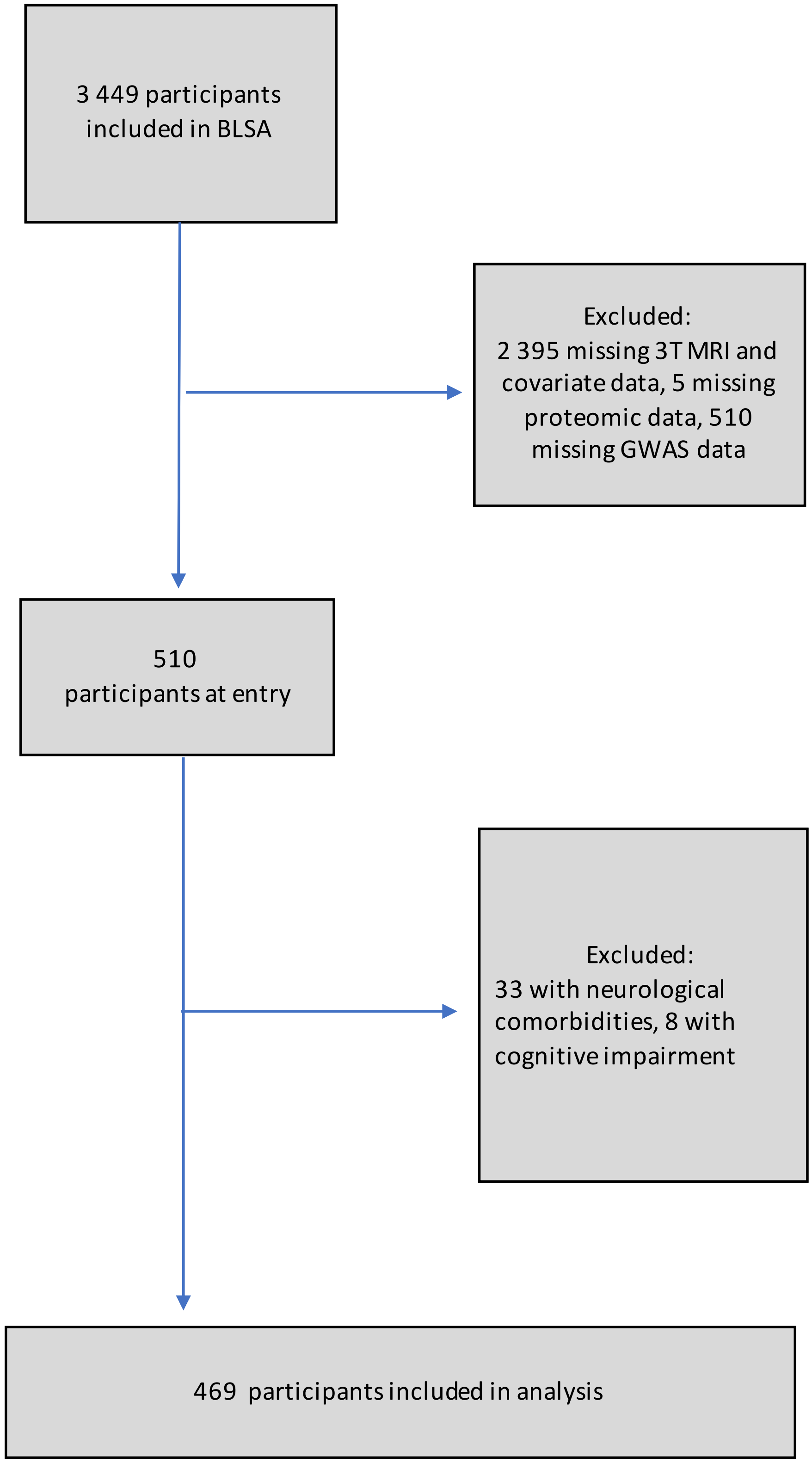

Supplementary Figure 10. Flow chart of participant selection for pQTL-MRI analyses in BLSA.

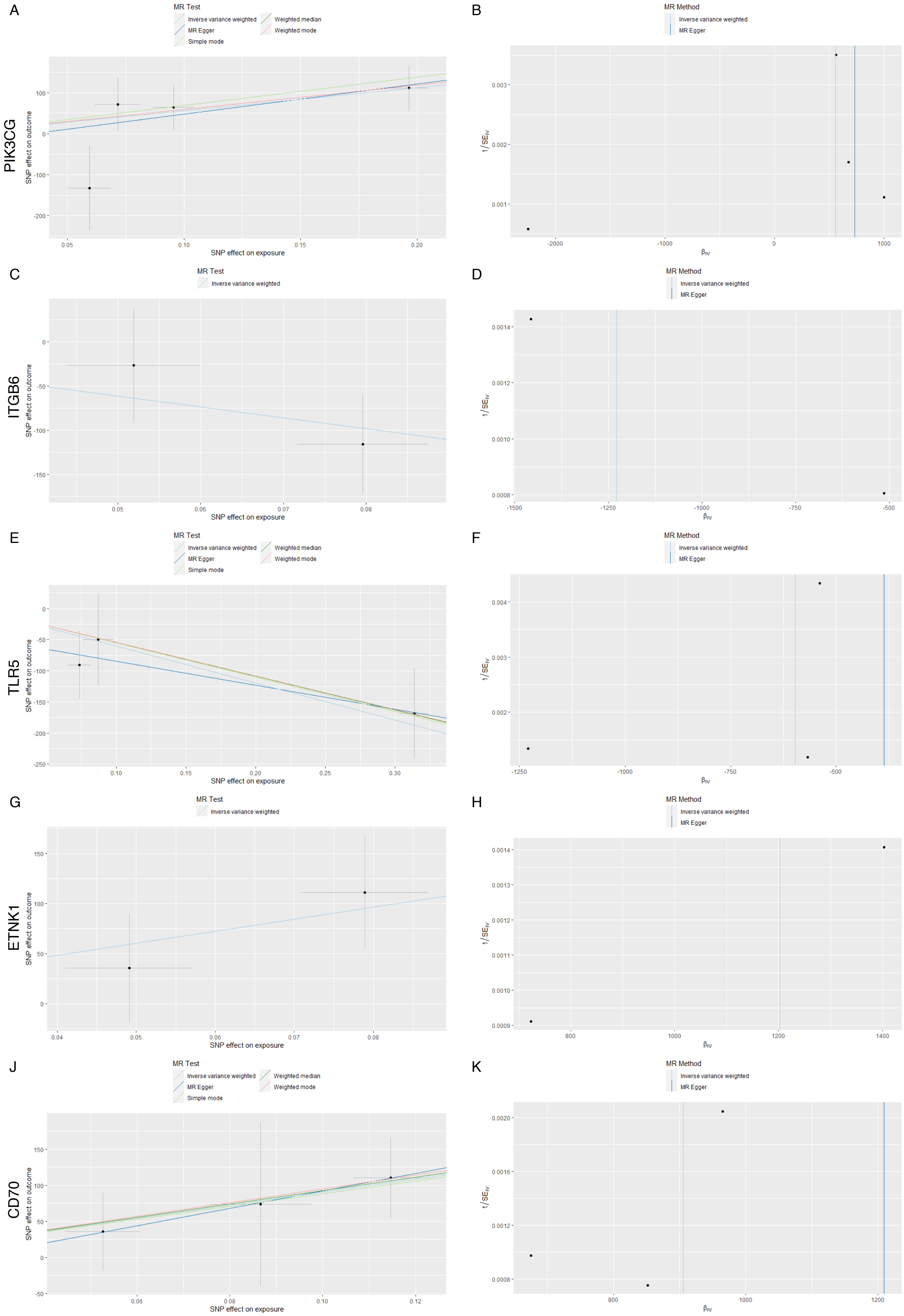

Supplementary Figure 11. Estimated effects of individual SNPs (**A**, **C**, **E**, **G**, **J**) and corresponding funnel plots (**B**, **D**, **F**, **H**, **K**) for two-sample MR analyses. Plasma pQTLs were obtained from deCODE Genetics (n=35,559). Longitudinal total brain volume summary statistics were obtained from an ENIGMA consortium GWAS (n=15,640). Data in **A, C, E, G, J** are presented as beta coefficients and 95% confidence intervals.

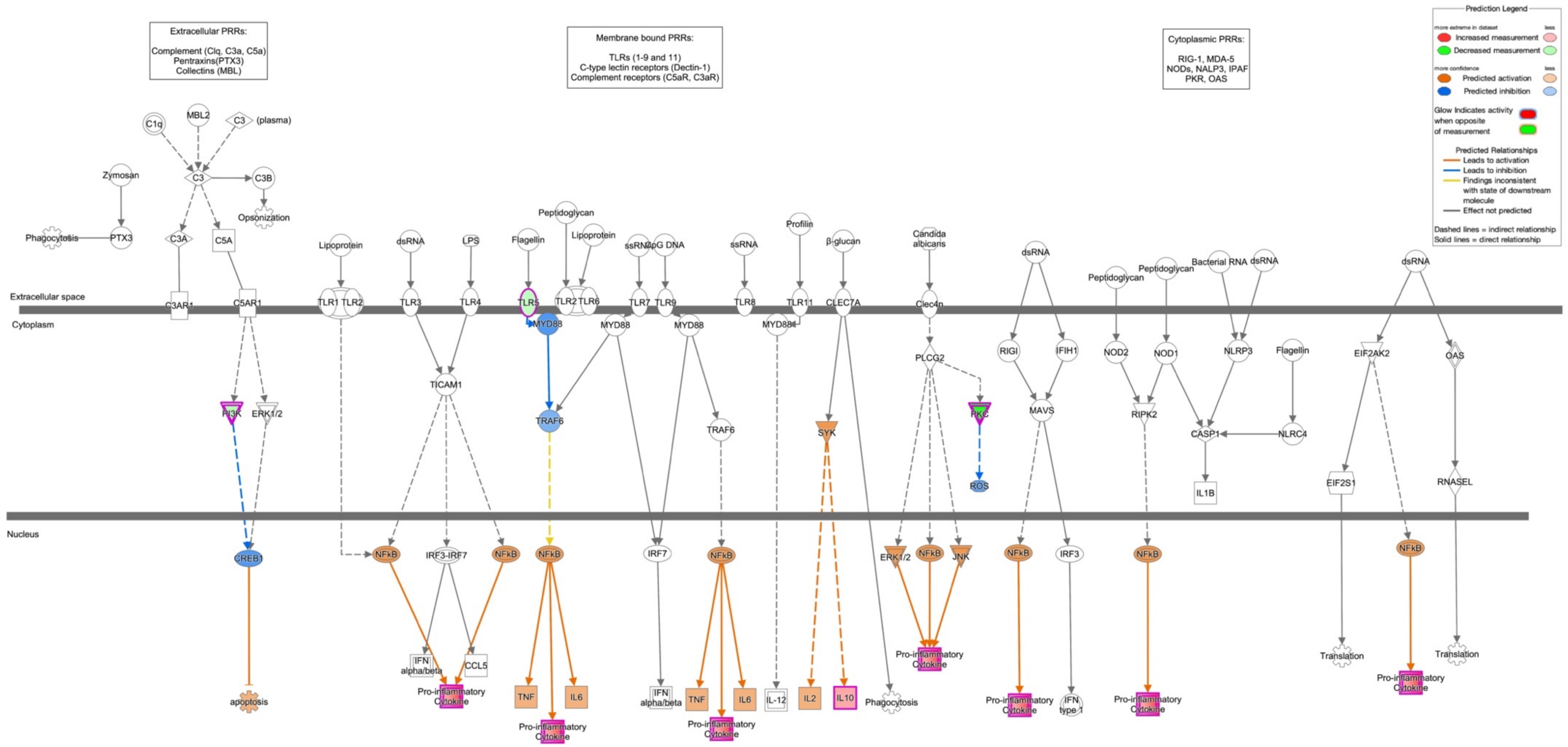

Supplementary Figure 12. Candidate protein-enriched canonical pathway (Role of Pattern Recognition Receptors in Recognition of Bacteria and Viruses).

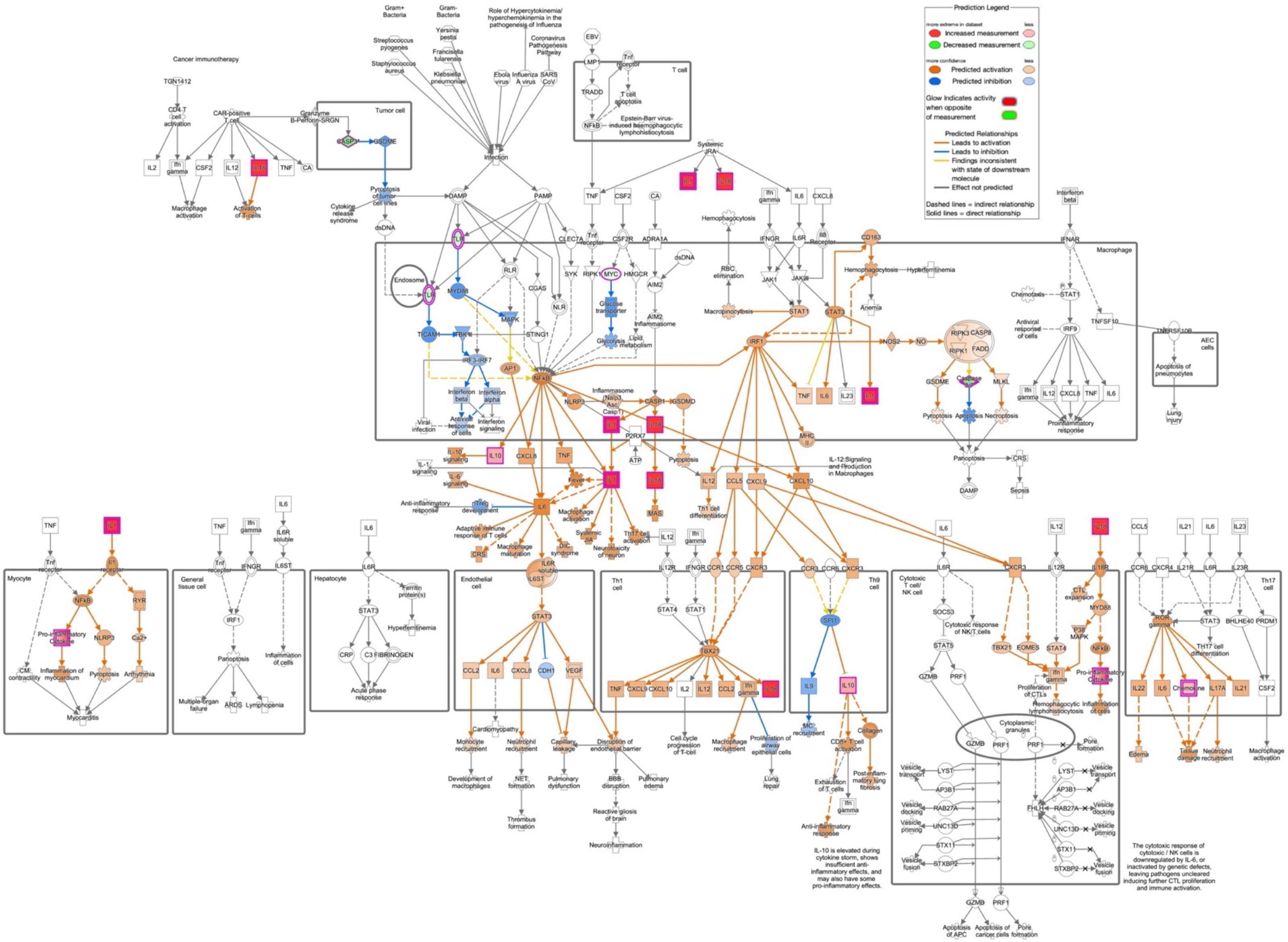

Supplementary Figure 13. Candidate protein-enriched canonical pathway (Pathogen Induced Cytokine Storm Signaling Pathway).

Supplementary references

1. Sipilä, P.N., et al. Hospital-treated infectious diseases and the risk of dementia: a large, multicohort, observational study with a replication cohort. Lancet Infect Dis 21, 1557-1567 (2021).
2. Gadd, D.A., et al. Integrated methylome and phenome study of the circulating proteome reveals markers pertinent to brain health. Nature Communications 13, 4670 (2022).
3. The Atherosclerosis Risk in Communities (ARIC) Study: design and objectives. The ARIC investigators. Am J Epidemiol 129, 687-702 (1989).
4. Walker, K.A., et al. Large-scale plasma proteomic analysis identifies proteins and pathways associated with dementia risk. Nature Aging 1, 473-489 (2021).
5. Knopman, D.S., et al. Mild Cognitive Impairment and Dementia Prevalence: The Atherosclerosis Risk in Communities Neurocognitive Study (ARIC-NCS). Alzheimers Dement (Amst) 2, 1-11 (2016).
6. Krämer, A., Green, J., Pollard, J., Jr. & Tugendreich, S. Causal analysis approaches in Ingenuity Pathway Analysis. Bioinformatics 30, 523-530 (2014).
7. Franceschini, A., et al. STRING v9.1: protein-protein interaction networks, with increased coverage and integration. Nucleic Acids Res 41, D808-815 (2013).
8. Yang, A.C., et al. A human brain vascular atlas reveals diverse mediators of Alzheimer's risk. Nature 603, 885-892 (2022).
