# Supplementary material for: Proteomics identifies potential immunological drivers of postinfection brain atrophy and cognitive decline
Source: Nat Aging. Author manuscript; Available in PMC 2024 Oct 11. (PMC11408246; doi:10.1038/s43587-024-00682-4)
Supplement: Supp information 2 [file NIHMS2021124-supplement-Supp_information_2.pdf]

nature portfolio

Corresponding author(s): Keenan A Walker

Last updated by author(s): 05/18/2024

Reporting Summary

Nature Portfolio wishes to improve the reproducibility of the work that we publish. This form provides structure for consistency and transparency in reporting. For further information on Nature Portfolio policies, see our Editorial Policies and the Editorial Policy Checklist.

Statistics

For all statistical analyses, confirm that the following items are present in the figure legend, table legend, main text, or Methods section.

n/a	Confirmed	
☐	☒	The exact sample size (n) for each experimental group/condition, given as a discrete number and unit of measurement
☐	☒	A statement on whether measurements were taken from distinct samples or whether the same sample was measured repeatedly
☐	☒	The statistical test(s) used AND whether they are one- or two-sided<br>*Only common tests should be described solely by name; describe more complex techniques in the Methods section.*
☐	☒	A description of all covariates tested
☐	☒	A description of any assumptions or corrections, such as tests of normality and adjustment for multiple comparisons
☐	☒	A full description of the statistical parameters including central tendency (e.g. means) or other basic estimates (e.g. regression coefficient) AND variation (e.g. standard deviation) or associated estimates of uncertainty (e.g. confidence intervals)
☐	☒	For null hypothesis testing, the test statistic (e.g. F, t, r) with confidence intervals, effect sizes, degrees of freedom and P value noted<br>*Give P values as exact values whenever suitable.*
☒	☐	For Bayesian analysis, information on the choice of priors and Markov chain Monte Carlo settings
☒	☐	For hierarchical and complex designs, identification of the appropriate level for tests and full reporting of outcomes
☐	☒	Estimates of effect sizes (e.g. Cohen's d, Pearson's r), indicating how they were calculated

Our web collection on statistics for biologists contains articles on many of the points above.

Software and code

Policy information about availability of computer code

Data collection	Cohort data was derived from the BLSA study using R (4.2.2). Proteomic measurements were outsourced to the vendor, SomaLogic Inc, Boulder Co USA. Analyses were restricted to the Inflammation and Immune Response Panel from the larger SomaScan v4.1 assay.
Data analysis	Analyses were performed using R version 4.2.2 (linear mixed effects models used nlme package (version 3.1.162) and Stata/MP version 17, two-sample MR analyses were performed using the TwoSampleMR (version 0.5.6), MendelianRandomization (version 0.6.0) packages), and MR-Presso (version 1.0). Additional packages included data.table (version 1.15), tidyverse (version 2.0), ggplot2 (version 3.5.1).LD pruning used the PLINK clumping algorithm available through the TwoSampleMR R package (version 0.5.6). Pathway analyses were conducted using Ingenuity Pathway Analysis (Qiagen Inc; version 01-22-01). Graphs were generated in R, Graphpad Prism (version 9.3.1), and the Biorender platform (https://www.biorender.com; version 0.1). Code is available through GitHub at /dugganmr/duggan-infections and /pyrysipila/sipila-infections-dementia-2.

For manuscripts utilizing custom algorithms or software that are central to the research but not yet described in published literature, software must be made available to editors and reviewers. We strongly encourage code deposition in a community repository (e.g. GitHub). See the Nature Portfolio guidelines for submitting code & software for further information.

Data

Policy information about availability of data

All manuscripts must include a data availability statement. This statement should provide the following information, where applicable:

- Accession codes, unique identifiers, or web links for publicly available datasets
- A description of any restrictions on data availability
- For clinical datasets or third party data, please ensure that the statement adheres to our policy

All data generated in the current study are included in this article (and its extended data figures, supplementary figures or supplementary tables), available upon reasonable request, or available in an online public repository. Researchers are welcome and encouraged to request use of BLSA data for scientific projects. Anonymized data not published within this article may be shared upon request from qualified investigators for purposes of replicating procedures and findings. Researchers who wish to use BLSA data are encouraged to develop a pre-analysis plan that can be submitted for approval (https://blsa.nia.nih.gov/how-apply). BLSA proteomic data have been deposited via the Alzheimer's Disease Data Initiative as part of the participation in the Global Neurodegeneration Proteomics Consortium (https://www.neuroproteome.org). Data, protocols, and other metadata of the UK Biobank are available to the scientific community upon request in accordance with the UK Biobank data sharing policy (https://www.ukbiobank.ac.uk/enable-your-research/apply-for-access). In the Finnish cohort studies, linked health records require separate permission from the Finnish Institute of Health and Welfare and Statistics Finland (https://thl.fi/en/web/thlfi-en/statistics-and-data/data-and-services/research-use-and-data-permits; https://tilastokeskus.fi/tup/mikroaineistot/aineistojen-yhdistaminen_en.html). ARIC proteomic data is available through the NHLBI Biologic Specimen and Data Repository Information Coordinating Center (https://biolincc.nhlbi.nih.gov/studies/aric/). Additional requests for clinical or proteomic data from individual investigators may be submitted to the ARIC steering committees and will be reviewed to ensure that data can be shared without compromising patient confidentiality or breaching intellectual property restrictions. Participant-level demographic, clinical and proteomic data may be partially restricted based on previously obtained participant consent. Data sharing restrictions may also be applied to ensure consistency with confidentiality or privacy laws and considerations (https://sites.cscc.unc.edu/aric/). Datasets of transcript expression levels for cell types were obtained through the Human Protein Atlas (https://www.proteinatlas.org/).

Research involving human participants, their data, or biological material

Policy information about studies with human participants or human data. See also policy information about sex, gender (identity/presentation), and sexual orientation and race, ethnicity and racism.

Reporting on sex and gender	Our main analyses are not stratified by sex or gender as this was not the focus of our research questions. Sex (self-reported) was used as a covariate throughout all analyses.
Reporting on race, ethnicity, or other socially relevant groupings	The primary sample included 982 cognitively normal participants (age=65.4 yrs. [SD=14.9]; 55.2% female; 66.9% white). Sample descriptions according to each analyses are reported in sTables 2, 6, 8, 11, 15, 18, 21, 23, 25 and 27.
Population characteristics	Data were collected as part of the Baltimore Longitudinal Study of Aging, an ongoing longitudinal study designed to assess physical and cognitive measures in a cohort of community-dwelling volunteers. Sample characteristics of the primary analyses were as follows: age=65.4 yrs. [SD=14.9]; 55.2% female; 66.9% white. A complete description of all participants groups and all demographic, physiological and comorbid data are provided in the supplementary tables. For more information, see the BLSA study website (https://www.blsa.nih.gov) and references:<br><br>Shock, N.W., et al. Normal human aging: The Baltimore longitudinal study of aging, (National Institutes of Health, Washington, D.C., 1984<br><br>Resnick, Susan M., et al. "Longitudinal magnetic resonance imaging studies of older adults: a shrinking brain." Journal of Neuroscience 23.8 (2003): 3295-3301.
Recruitment	Community-dwelling volunteers were recruited from the city and surrounding areas of Baltimore, Maryland.
Ethics oversight	The BLSA protocol was approved by the Institutional Review Board of the National Institute of Environmental Health Science, National Institutes of Health (03AG0325). All participants gave written informed consent prior to participation and deidentified data were used for analyses. The UK Biobank study was approved by the National Health Service National Research Ethics Service (11/NW/0382). All participants gave informed consent. The Finnish Public Sector study (FPS) was approved by the ethics committee of the Hospital District of Helsinki and Uusimaa (HUS/1210/2016), the Health and Social Support study (HeSSup) was approved by the ethics committee of Turku University Hospital and the Finnish Population Register Centre (VRK 2605/410/14), and the Still Working study (STW) was approved by the ethics committee of the Finnish Institute of Occupational Health. All participants gave informed consent. In FPS, there were additional participants from whom only de-identified register data were collected and thus no consent was required. ARIC study protocols were approved by institutional review boards at each participating center: University of North Carolina at Chapel Hill, Chapel Hill, NC; Wake Forest University, Winston-Salem, NC; Johns Hopkins University, Baltimore, MD; University of Minnesota, Minneapolis, MN; and University of Mississippi Medical Center, Jackson, MS. All ARIC participants gave written informed consent at each study visit; proxies provided consent for participants who were judged to lack capacity.

Note that full information on the approval of the study protocol must also be provided in the manuscript.

Field-specific reporting

Please select the one below that is the best fit for your research. If you are not sure, read the appropriate sections before making your selection.

☒ Life sciences ☐ Behavioural & social sciences ☐ Ecological, evolutionary & environmental sciences

For a reference copy of the document with all sections, see nature.com/documents/nr-reporting-summary-flat.pdf

Life sciences study design

All studies must disclose on these points even when the disclosure is negative.

Sample size	The sample sizes for the primary analyses and secondary, external analyses were derived based on available data in the BLSA and corresponding cohorts (e.g., UK Biobank, ARIC). Sample sizes are similar to those reported in previous publications (e.g., Duggan et al., 2023; Sipila et al., 2022; Walker et al., 2023). No power calculation was conducted a priori for the current analyses. With over 900 participants for the primary analyses and for each of the external analyses, we are well-powered to detect meaningful associations between predictors (e.g., infection exposure) and outcomes (e.g., plasma protein abundance).
Data exclusions	The sample used for primary analyses was selected based on available International Classification of Diseases, Ninth Revision (ICD9) codes, and MRI, SomaScan v4.1 proteomic, Simoa biomarker, cognitive task or GWAS data, as well as no neurological conditions that could affect brain structure or function (e.g., strokes, seizures) and no cognitive impairment at baseline or follow-up (i.e., dementia, MCI, impaired but not MCI).
Replication	A direct replication of primary analyses (i.e., the effect of infection exposures on brain volume changes over time) is not possible at this point because there are no comparable datasets (i.e., no other cohorts have both clinically-adjudicated infection exposures and longitudinal 3T MRI scans on 900+ participants).
Randomization	Randomization was not applicable to our study design. This was an observational cohort study conducted on data that was already collected and stored. As such, no allocation to experimental groups was performed.
Blinding	3T MRI scans were obtained by the BLSA clinical research core who were blinded to status of the sample. Protein measurement was outsourced to the SomaLogic research team who were also blinded to status of the sample. Investigators were blinded during data collection and throughout analyses.

Reporting for specific materials, systems and methods

We require information from authors about some types of materials, experimental systems and methods used in many studies. Here, indicate whether each material, system or method listed is relevant to your study. If you are not sure if a list item applies to your research, read the appropriate section before selecting a response.

Materials & experimental systems

n/a	Involved in the study	
☒	☐	Antibodies
☒	☐	Eukaryotic cell lines
☒	☐	Palaeontology and archaeology
☒	☐	Animals and other organisms
☒	☐	Clinical data
☒	☐	Dual use research of concern
☒	☐	Plants

Methods

n/a	Involved in the study	
☒	☐	ChIP-seq
☒	☐	Flow cytometry
☐	☒	MRI-based neuroimaging

Plants

Seed stocks	NA
Novel plant genotypes	NA
Authentication	NA

Magnetic resonance imaging

Experimental design

Design type	structural MRI
Design specifications	T1-weighted magnetization-prepared rapid gradient echo (MPRAGE) scans were acquired on a 3-T Philips Achieva (repetition time [TR] = 6.8 ms, echo time [TE] = 3.2 ms, flip angle = 8°, image matrix = 256 × 256, 170 slices, pixel size = 1 × 1 mm, slice thickness = 1.2 mm)
Behavioral performance measures	Not applicable. Participants were not asked to perform tasks or behaviors during scans.

Acquisition

Imaging type(s)	structural
Field strength	3T
Sequence & imaging parameters	A fully automated pipeline was used for processing structural MRIs. A multi-atlas skull stripping algorithm using a large set of reference atlases was first applied for robust and accurate extraction of the brain tissues. In order to maintain longitudinal consistency, the brain mask at each scan time point was combined with a probabilistic brain mask that was propagated from the baseline image. Skull stripped T1 images were corrected for intensity inhomogeneities using FAST. Each scan was segmented into a set of anatomical regions of interest (ROIs) using a multi-atlas label fusion method, MUSE, which obtained state-of-the-art accuracy in independent evaluations against benchmark methods. In this framework, multiple atlases with semi-automatically extracted ground-truth ROI labels are individually warped through deformable registration to the target image using two different registration methods, and the warped labels are fused into a consensus segmentation. For longitudinal scans, a pseudo-4D approach was used by propagating atlases that were warped to the baseline image space into each follow-up time point, thus imposing a more unified registration path before the fusion of reference labels.A convolutional deep learning based method, DeepMRSeg, was used for segmenting a mask of the intra-cranial area. The proposed method is built upon the UNet architecture with the convolutional layers in the network replaced by an Inception ResNet architecture. The segmentation model was trained on a large multi-site dataset with manually verified ground-truth masks that were obtained using T1 and T2 weighted scans. The model was applied on each raw T1-weighted image. Intra-cranial volume was estimated from the output mask, and it was used in subsequent analysis steps for the adjustment of imaging values against inter-individual differences in head size.
Area of acquisition	whole brain

Diffusion MRI ☐ Used ☒ Not used

Preprocessing

Preprocessing software	Skull stripped T1 images were corrected for intensity inhomogeneities using FAST.
Normalization	Brain volumes was measured on MPRAGE images in subject space then mapped to MNI152 using ANTs.
Normalization template	MNI152
Noise and artifact removal	The BLSA Neuroimaging Study's Clinical Core reviewed image quality, image processing procedures, and image segmentation as a quality control check. Preprocessing was used to address motion artifacts.
Volume censoring	None. This study used structural, rather than functional, MRI.

Statistical modeling & inference

Model type and settings	Linear mixed-effects models were used to examine associations of infections with longitudinal rates of change in brain volumes. In addition to adjusting for intracranial volume, models included the following covariates: baseline age, sex, race, education, APOEε4, comorbidity index and the interactions of age, sex, race, education, APOEε4 and comorbidity index with time. Random effects of intercept and time with unstructured covariance were included.
Effect(s) tested	Associations of infections with longitudinal brain volume changes in primary analyses; secondary analyses also examined associations of plasma proteins with longitudinal brain volume changes.

Specify type of analysis: ☐ Whole brain ☒ ROI-based ☐ Both

Anatomical location(s)	Analyses examined standardized values of total brain, gray matter, white matter and lobar volumes (Frontal, Parietal, Occipital, Temporal), as well as an AD Signature Region volume (i.e., the combined volume of hippocampus, parahippocampal gyrus, entorhinal cortex, posterior cingulate gyrus, precuneus, and cuneus). If an infection was significantly associated with a primary region of interest, we performed secondary analyses on lobar white/gray matter volumes. Standardized volumes of all 48 MUSE labeled regions were used to explore changes related pQTLs (see below for further description of pQTL analyses).

Statistic type for inference

(See Eklund et al. 2016)

We did not conduct voxel-level analysis.

Correction

We did not conduct voxel-level analysis.

Models & analysis

n/a	Involved in the study
☒	☐ Functional and/or effective connectivity
☒	☐ Graph analysis
☒	☐ Multivariate modeling or predictive analysis
